# Supplementary material for: Expression of RUNX1-ETO Rapidly Alters the Chromatin Landscape and Growth of Early Human Myeloid Precursor Cells
Source: Cell Rep. 2020 May 26;31(8):107691. doi: 10.1016/j.celrep.2020.107691 (PMC7262600; doi:10.1016/j.celrep.2020.107691)
Supplement: Document S1. Figures S1–S6 and Table S1 [file mmc1.pdf]

Cell Reports, Volume *31*

Supplemental Information

Expression of RUNX1-ETO Rapidly Alters the Chromatin Landscape and Growth of Early Human Myeloid Precursor Cells

Monica Nafria, Peter Keane, Elizabeth S. Ng, Edouard G. Stanley, Andrew G. Elefanty, and Constanze Bonifer

Figure S1

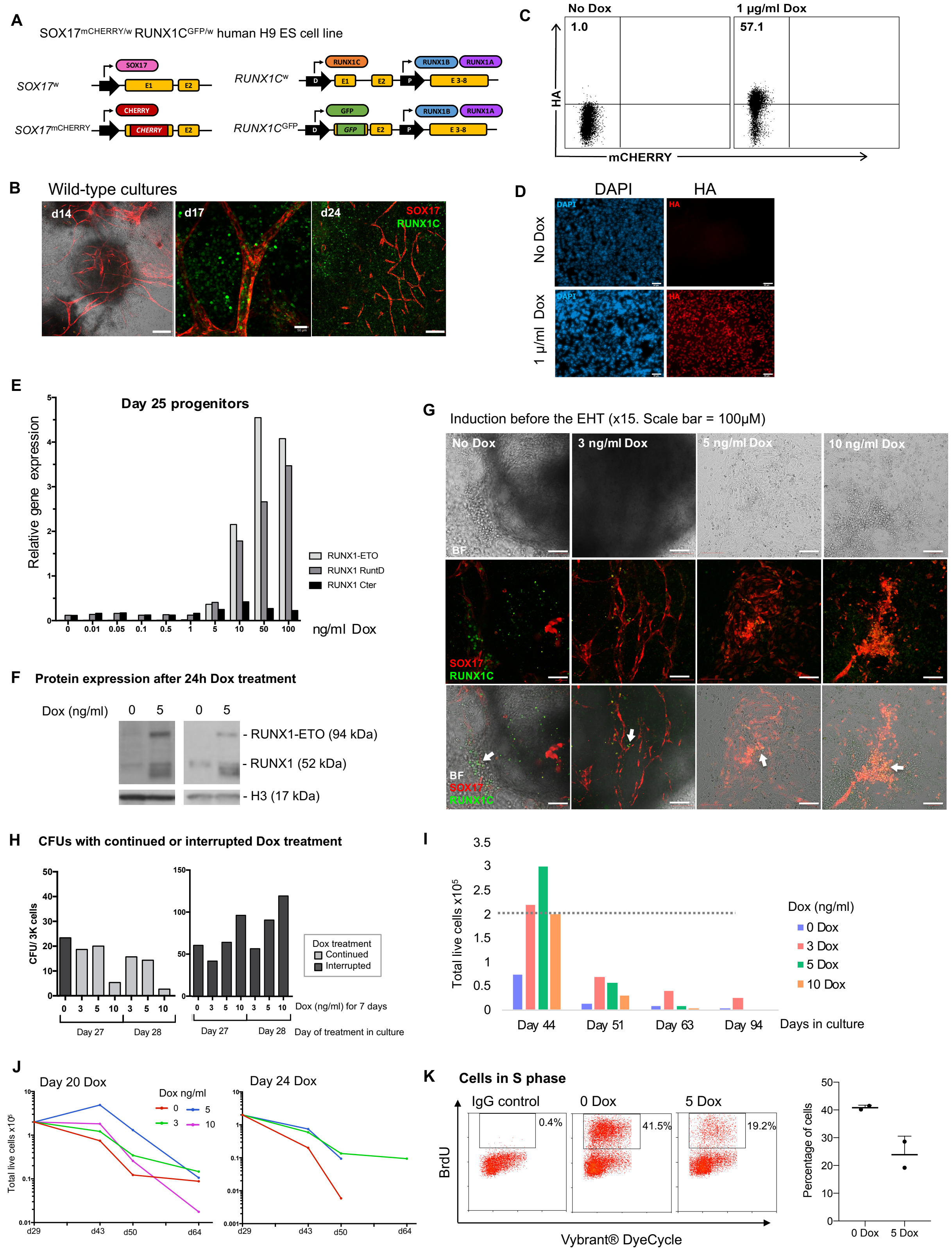

FIGURE S1 (Related to Figures 1 and 2). Expression of *RUNX1-ETO* leads to a reversible differentiation and growth arrest of human early hematopoietic progenitor cells

A) Schematic representation of the targeted alleles in the $SOX17^{mCHERRY/w}$ $RUNX1C^{GFP/w}$ human H9 ES dual reporter cell line. Left: Wild-type and targeted alleles in the *SOX17* locus, with mCHERRY sequence inserted into exon 1. Right: Wild-type and targeted alleles in the *RUNX1* locus. GFP sequence was inserted into exon 1, resulting in expression of GFP from the distal (D) promoter and RUNX1B and RUNX1A isoforms from the proximal (P) promoter within the same allele. Promoters and exons are shown with black arrows and yellow boxes, respectively. Protein products generated from each allele are represented with ovals.

B) RUNX1C+ hematopoietic progenitors emerge from cell clusters located within vascular structures of SOX17+ hemogenic endothelium after the EHT in wild-type EB cultures. Confocal images of EB cultures from the $SOX17^{mCHERRY/w}$ $RUNX1C^{GFP/w}$ human H9 ES double reporter cell line at several days during differentiation, as indicated. *SOX17* (mCHERRY) expression marks arterial structures and *RUNX1C* (GFP) marks hematopoietic progenitors. Scale bar: 200 µm (d14, d24), 50 µm (d17). Fluorescence and brightfield channels are merged at d14 image. At d14: EBs appear as opaque round structures surrounded by a stromal layer containing SOX17+ hemogenic endothelium and arterial structures. At d17: RUNX1C+ blood progenitors are generated from SOX17+ hemogenic endothelium, mimicking structures observed during embryonic AGM hematopoiesis. At d24: progenitors have detached from the endothelium and gone in suspension to further grow and differentiate.

C) Intracellular flow cytometry analysis showing RUNX1-ETO induction upon addition of 1 µg/ml Dox for two days in a pooled population of puromycin-resistant cells after transfection. Detection using an anti-HA tag DyLight® 650-conjugated antibody.

D) Immunofluorescence assay showing RUNX1-ETO induction upon addition of 1 µg/ml Dox for two days in a single-sorted clone. Images are of clone #18 and representative of six single-sorted clones. Cell nuclei are stained with DAPI (blue) and cells expressing HA-*RUNX1-ETO* are detected with an anti-HA antibody (red). Fluorescence channels are merged on right panels. Scale bar: 50 µm.

E) *RUNX1* and *RUNX1-ETO* gene expression in response to Dox treatment for 24h on d25 hematopoietic progenitors. Primers were designed to amplify: the translocation breakpoint (RUNX1-ETO), the DNA-binding domain present in both gene products (RUNX1 RuntD) and the carboxy-terminal domain present only in endogenous RUNX1 (RUNX1 C-ter). Gene expression was normalized to that of GAPDH.

F) RUNX1 and RUNX1-ETO protein expression measured by Western Blotting in nuclear extracts from floating progenitors uninduced (0 Dox) and induced with Dox (5 ng/ml) at day 21 for 24h. The figure shows two biological replicates. RUNX1 and RUNX1-ETO were detected using the same antibody, which binds to the RUNX1 homology domain. H3 protein expression was measured as a loading control.

G) RUNX1-ETO induction before the EHT disrupts the vascular organization and disrupts blood formation. Confocal images of combined Z-stack layers from d16 hematopoietic differentiation cultures with RUNX1-ETO induced from d10 (before the EHT) using 3, 5 or 10 ng/ml Dox. White arrows in the 0 Dox condition indicate emerging RUNX1C-/+ blood progenitors. Arrows in the Dox-treated samples indicate aberrant phenotypes including reduced numbers of emerging progenitors (3 ng/ml Dox), disorganized vasculature (5 ng/ml Dox) and progenitors co-expressing *RUNX1C* and *SOX17* (10 ng/ml Dox). Brightfield (top), fluorescence (middle) and merged field channels (bottom) are shown. Scale bars: 100 µm. SOX17 (mCHERRY, red) and RUNX1C (GFP, green).

H) RUNX1-ETO causes a reversible proliferation block (Related to Figure 2A). Colony-forming unit (CFU) assays of definitive progenitors from EB cultures treated with Dox during 7 days at day 27 and day 28. For the CFU assays, progenitors were plated in methylcellulose in triplicate at a concentration of 3,000 live cells/well in either presence (continued treatment, light grey) or absence (interrupted treatment, dark grey) of Dox. Individual graphs correspond to different biological replicates.

I) Previously induced progenitor cells show an initial growth response and increased survival compared to uninduced cells (Related to Figure 2B). EB cultures were treated at d22 with 0, 3, 5 or 10 ng/ml Dox for 7 days and floating progenitors were harvested and subjected to serial replating assays with continued Dox treatment. The graph displays the cell growth data represented in Figure 2B in absolute values ($x10^5$) of the total live cells. The dotted line represents the number of progenitors plated at the start of the replating assay.

J) Low levels of RUNX1-ETO induction increases the survival of a subset of progenitor cells. Additional examples to data shown in Figure 2B. Replating assays of hematopoietic progenitors from cultures treated at d20 or d24 with different Dox concentrations, showing two representatives each of three independent experiments. Floating hematopoietic cells were plated at 2 $x10^5$ cells/well in the correspondent Dox concentration and cell numbers were measured weekly at three time points, as indicated. On d24 graph, only 3-dox treated cells were able to survive over 28 days in the replating assays.

K) RUNX1-ETO produces a cell cycle arrest in the G1 phase. Flow cytometry plots showing cell cycle kinetics of wild-type and RUNX1-ETO-induced progenitor cells. EB cultures were induced at d21 with 5 ng/ml Dox for 4 days and then were pulse-labelled with 25 µM BrdU for 3.5h. Non-adherent cell progenitors were fixed and stained with FITC-conjugated anti-BrdU antibody and Vybrant-DyeCycle. DNA content and cell cycle distribution were analyzed by flow cytometry. Boxed cells represent cells that have entered the S-phase of the cell cycle during the BrdU incubation. FITC IgG control is shown. Graph on the right shows the percentage of cells in S-phase corresponding to two biological replicates (represented as dots) and the median values (represented with a line).

Figure S2

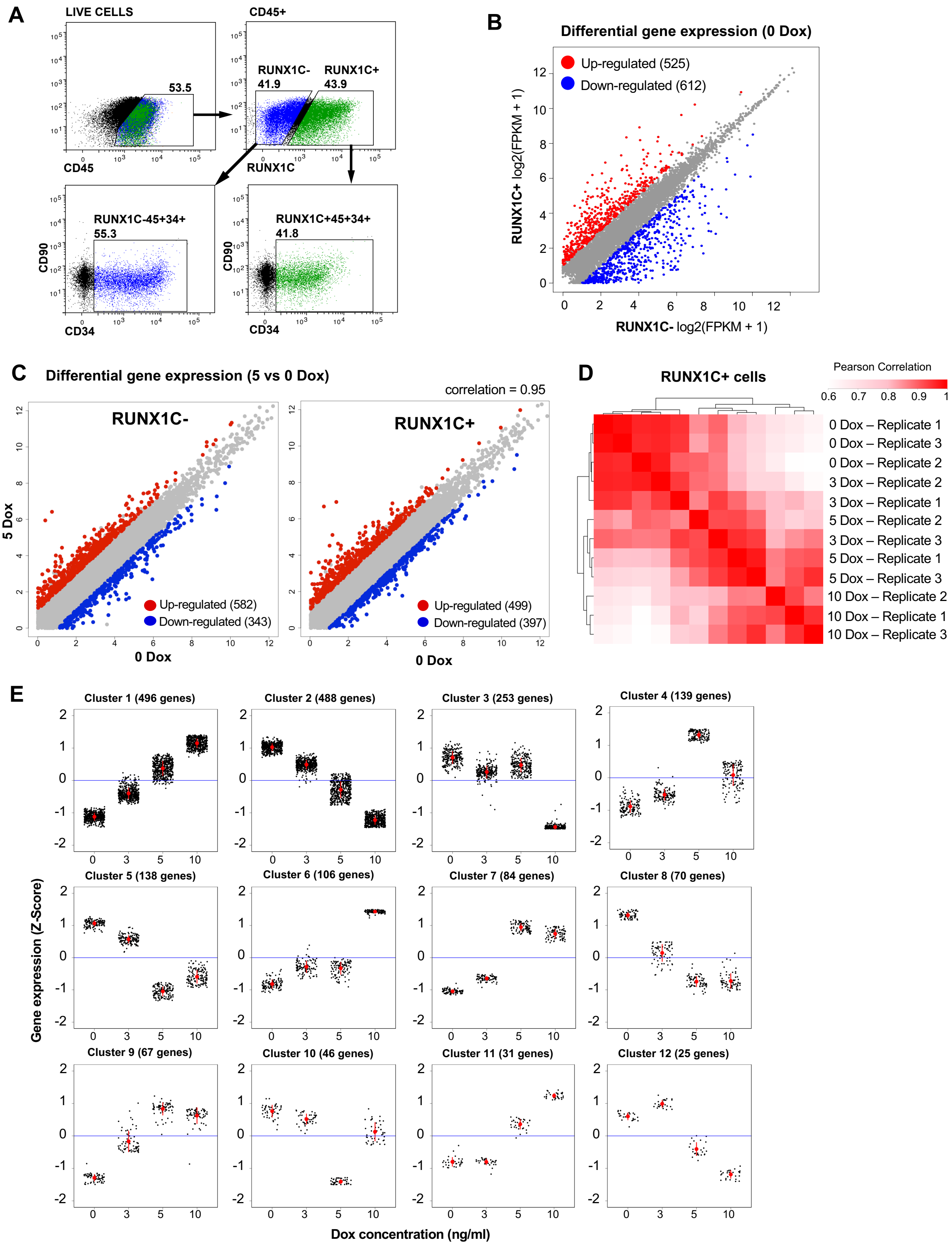

FIGURE S2 (Related to Figure 3). RUNX1-ETO induction leads to cell-type and dose-dependent changes in gene expression

A) Flow cytometry strategy for sorting of d21 cultures based on CD45 (BV), RUNX1C (GFP/FITC) and CD34 (Pe-Cy7) expression.

B) Clustering of gene expression RNA-Seq data by log2 fold FPKM +1 (fragments per kilobase of transcripts per million mapped reads) values of genes differentially expressed (two-fold change) after RUNX1-ETO induction using 5 ng/ml Dox in both RUNX1C- and RUNX1C+ (CD45+ CD34+) populations. Adjusted P value <0.05.
C) Clustering of gene expression RNA-Seq data by log2 fold FPKM +1 (fragments per kilobase of transcripts per million mapped reads) values of genes differentially expressed (two-fold change) after RUNX1-ETO induction using 5 ng/ml Dox in both RUNX1C- and RUNX1C+ (CD45+ CD34+) populations. Adjusted P value <0.05.
D) Hierarchical clustering using Pearson correlation coefficients of gene expression in CD45+ CD34+ RUNX1C+ progenitors upon 24-hour Dox exposure (0, 3, 5 or 10 ng/ml) from three biological replicates.
E) Co-variance analysis of gene expression RNA-Seq data by Z-score from CD45+ CD34+ RUNX1C+ sorted progenitor cells upon RUNX1-ETO induction with 3, 5 or ng/ml Dox for 24h, showing 12 clusters/groups of genes with differential expression response to the level of RUNX1-ETO induction. Number of genes comprised on each cluster are indicated. Black dots represent transcript levels for each individual gene. Red dots and bars represent the mean and standard deviation.

Figure S3

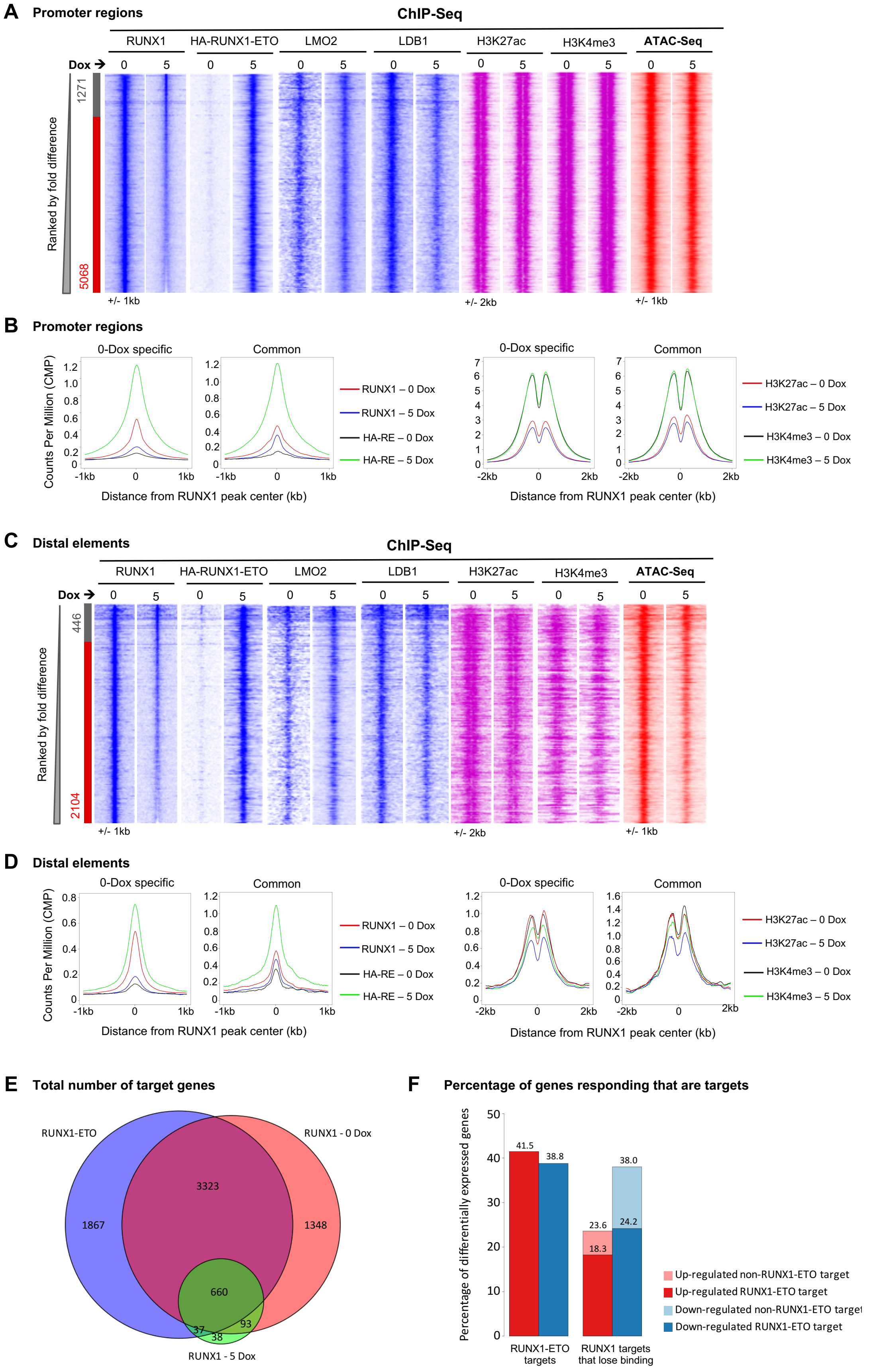

FIGURE S3 (Related to Figures 4 and 5). RUNX1-ETO blocks the binding of RUNX1 at distal elements and leads to down-regulation of RUNX1 (but not RUNX1-ETO) target genes

A and C) Comparison of RUNX1 binding at promoter (A) and distal (C) regions from ChIP-Seq in 0 and 5 Dox-treated CD34+ populations ranked by fold difference, considering peaks with enrichments greater than 2-fold between samples to be specific. Sample-specific sites and number of peaks are indicated alongside, being: red the 0-Dox specific and grey the shared peaks. ChIP-Seq enrichment for (HA)-RUNX1-ETO, LMO2, LDB1, H3K27ac and H3K4me3 in each sample and chromatin accessibility (ATAC-Seq) peaks are plotted along the same coordinates as the RUNX1 ChIP-Seq promoter (A) and distal element (C) peaks.
B and D) Average profiles for transcription factor (Top panels) and histone modification (Bottom panels) ChIP-Seq data centered on RUNX1 promoter (B) or RUNX1 distal element (D) -binding peaks in the 0 Dox-specific and common peaks.
E) Venn diagram of RUNX1-ETO and RUNX1 ChIP data showing overlap of binding events between the total number of genes targeted by (i) RUNX1-ETO, (ii) RUNX1 0 Dox (in uninduced cells) and (iii) RUNX1 after 5 Dox induction.
F) Graph depicting the percentage of differentially expressed (up- or down-regulated) genes that respond to RUNX1-ETO induction and are either RUNX1-ETO or RUNX1 targets.

Figure S4

A

Sequencing statistics - 0 Dox	
No. of cells sequenced	3,327
No. of cells passed QC	3,141
Median no. of reads per cell	75,452
Median no. of genes per cell	3,149

Sequencing statistics - 5 Dox	
No. of cells sequenced	3,814
No. of cells passed QC	3,669
Median no. of reads per cell	67,098
Median no. of genes per cell	3,166

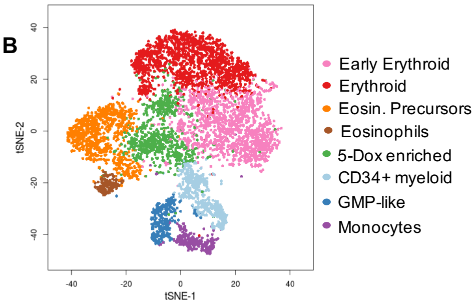

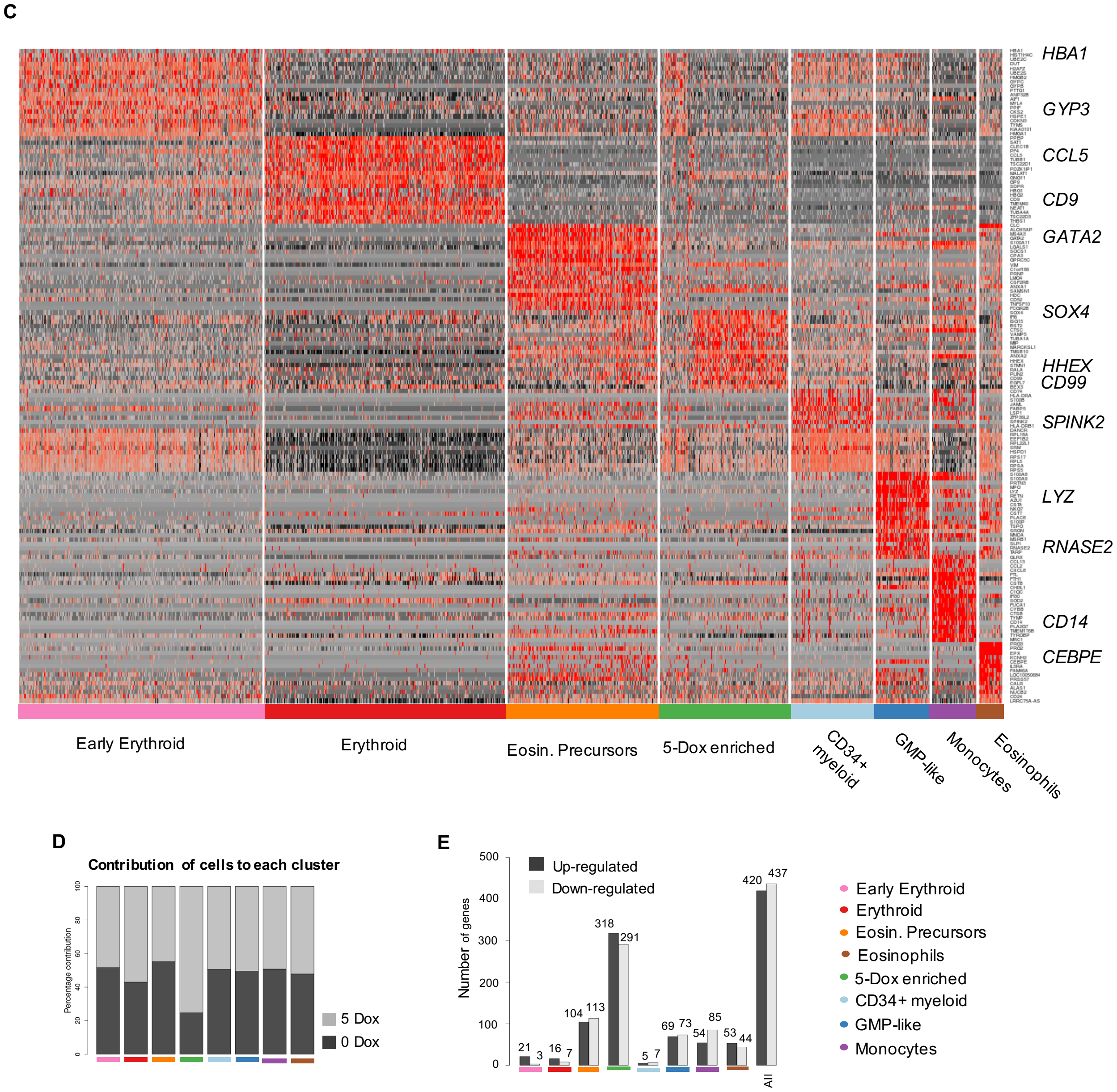

FIGURE S4 (Related to Figure 6). The CD45+CD34+RUNX1C+ population contains precursors from distinct blood lineages as well as multipotent cell progenitors

A) Table of the scRNA-Seq sequencing statistics showing the total number of sequenced cells, number of cells that passed the quality control (QC), and the median of reads and sequenced genes per cell for each 0 and 5 ng/ml Dox conditions.

B) Two-dimensional t-SNE maps displaying a total number of 7,135 CD45+ CD34+ RUNX1C+ sorted single cells from the combined data of 0 and 5 Dox treated cells, including identified cell populations based on expression of known marker genes.

C) Heatmap showing the expression of the top 20 marker genes specific to each cluster (same color coding as in (B)). Representative genes from each cluster are indicated.

D) Proportion bars showing the percentage of contribution of 0 and 5 ng/ml Dox dataset to each individual cell cluster.

E) Number of up- and down- regulated genes in each cell cluster upon treatment with 5 ng/ml Dox.

Figure S5

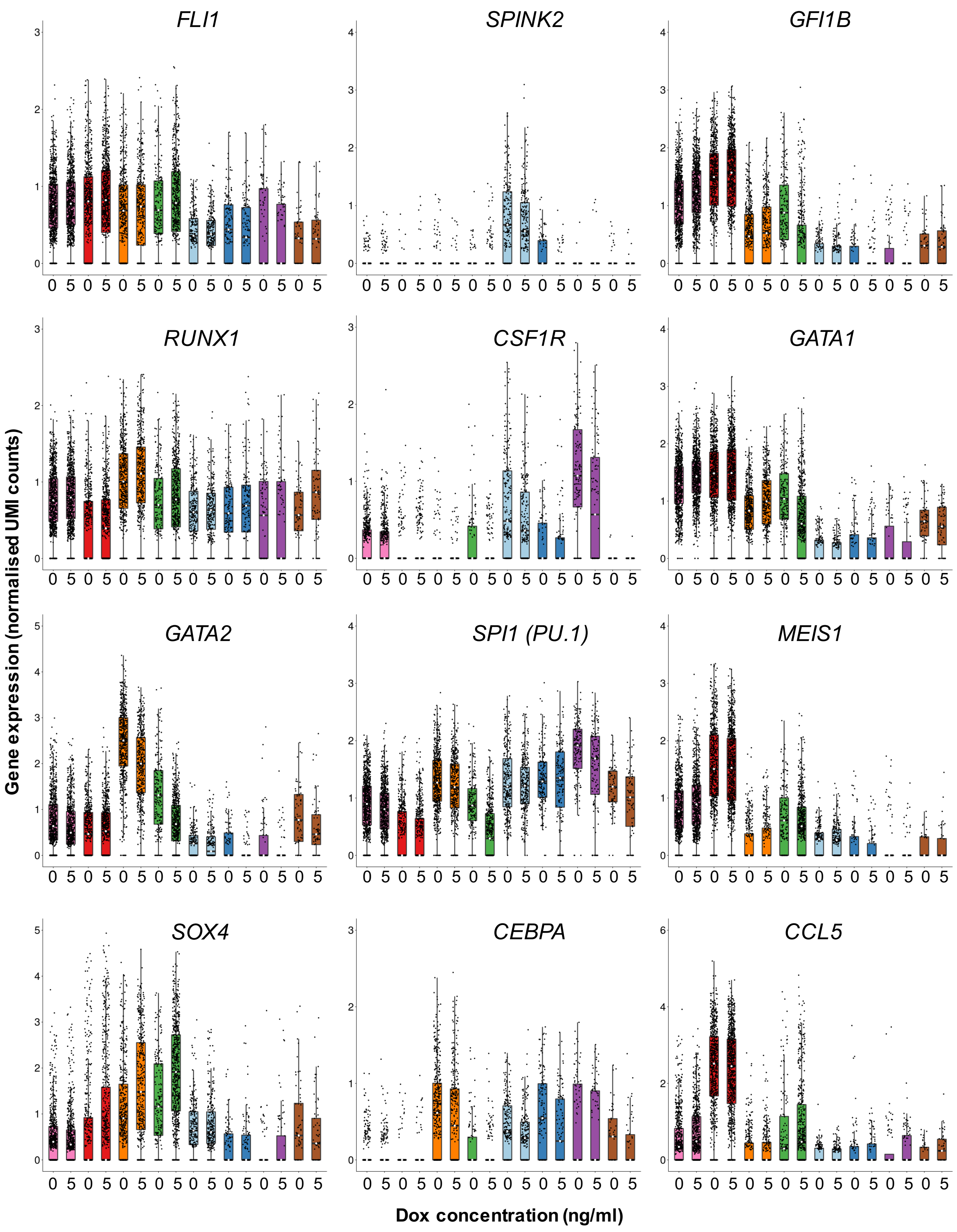

FIGURE S5 (Related to Figure 6). Induction of RUNX1-ETO results in the up-regulation of *SOX4* as well as down-regulation of important regulators of myelopoiesis in the 5-Dox enriched population

Box plots indicating expression levels of the individual marker genes represented in Fig 6D in the different ESC derived cell populations (color coded) and both datasets (0 and 5 Dox). Black dots represent transcript levels in an individual single cell. Boxes and bars represent the mean and standard deviation, respectively.

Figure S6

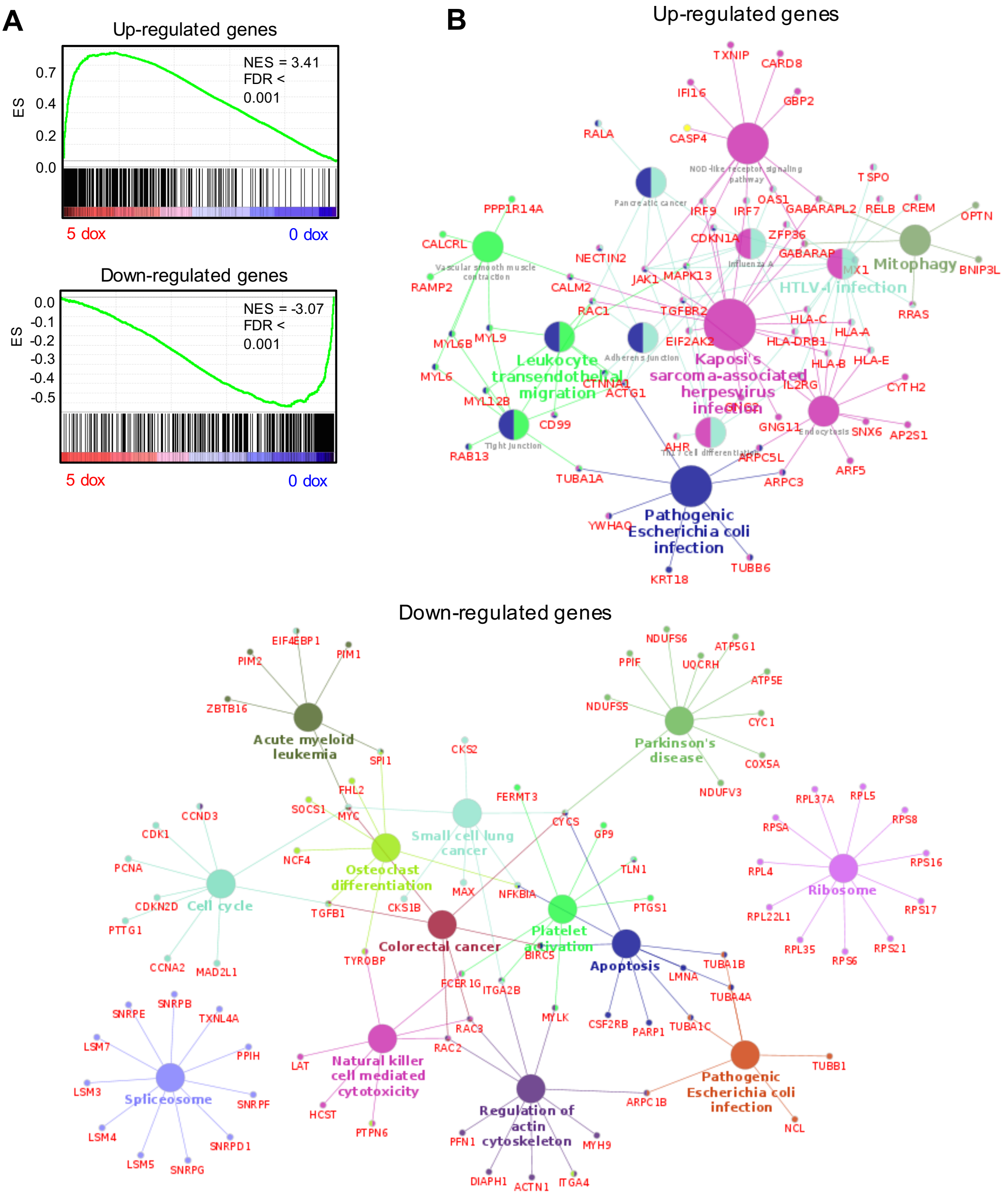

FIGURE S6 (Related to Figure 6). RUNX1-ETO induction yields a similar pattern of transcriptional dysregulation in the 5-Dox enriched and the 5 Dox-treated bulk progenitor populations

A) Gene Set Enrichment Analysis for correlation of up-regulated (top panel) and down-regulated (bottom panel) gene signatures between single-cell sorted cells and the CD45+ CD34+ RUNX1C+ bulk populations upon 5-Dox induction for 24 hours. ES, Enrichment Score; NES, Normalized Enrichment Score. FDR, False discovery rate.

B) Network diagram of KEGG pathways for up-regulated (above) and down-regulated (below) genes in the 5-Dox enriched cell cluster of CD45+ CD34+ RUNX1C+ sorted single cells upon 24-hours 5 ng/ml Dox treatment.

**Oligonucleotides**	**Forward**	**Reverse**
RUNX1-ETO cloning	TACCGTCGACCCGCCATGTACCCA TACGACGTCCCAGACTACGCTCGT ATCCCCGTAGATGCCAGCACGA	CGCAACGCGTCTACTAGCGAGGGGTTG TCTCTA
AAVS1 5' screen	GGACCACTTTGAGCTCTACT	TCCACGTCACCGCATGTTAG
AAVS1 3' screen	TGCCTGCTGACGCTCTTGACGATT	GAAGGATGCAGGACGAGAAA
Wild-Type screen	CCCCTATGTCCACTTCAGGA	CAGCTCAGGTTCTGGGAGAG
GAPDH	CCTGGCCAAGGTCATCCAT	AGGGGCCATCCACAGTCTT
RUNX1 C-ter	CCCTCAGCCTCAGAGTCAGAT	GGCAATGGATCCCAGGTAT
RUNX1 Runt Domain	AACAAGACCCTGCCCATCGCTTTC	CATCACAGTGACCAGAGTGCCAT
RUNX1-ETO junction	TCAAAATCACAGTGGATGGGC	CAGCCTAGATTGCGTCTTCACA

TABLE S1 (Related to Key Resources Table in STAR METHODS). List of primers generated in this study used for cloning, insertion of the transgene during cell line generation and gene expression analyses.
