# Supplementary material for: Expression of RUNX1-ETO Rapidly Alters the Chromatin Landscape and Growth of Early Human Myeloid Precursor Cells
Source: Cell Rep. 2020 May 26;31(8):107691. doi: 10.1016/j.celrep.2020.107691 (PMC7262600; doi:10.1016/j.celrep.2020.107691)
Supplement: Document S2. Article plus Supplemental Information [file mmc9.pdf]

Cell Reports

Article

Expression of RUNX1-ETO Rapidly Alters the Chromatin Landscape and Growth of Early Human Myeloid Precursor Cells

Graphical Abstract

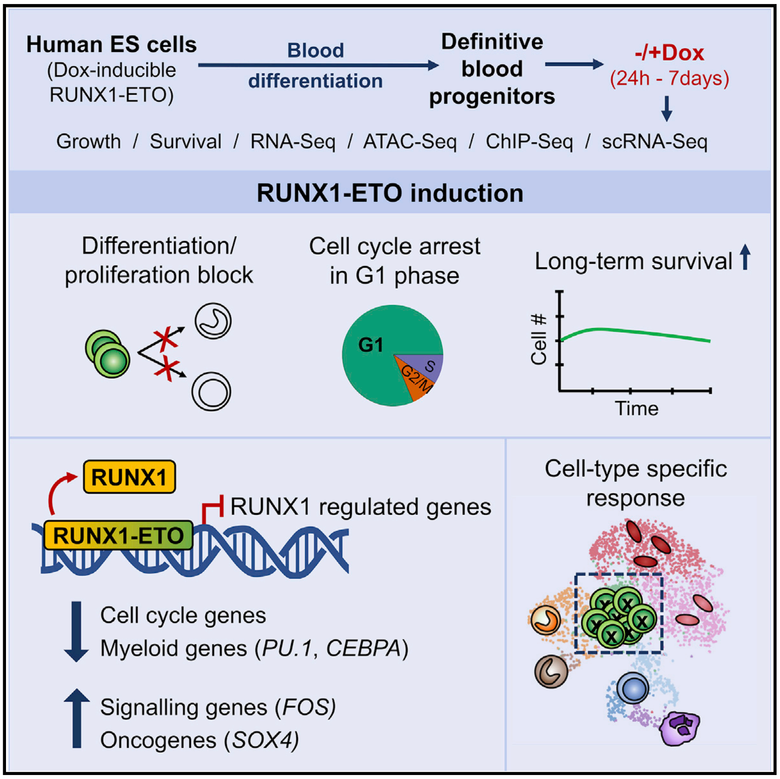

Highlights

- RUNX1-ETO reversibly arrests the growth of human ESC-derived early myeloid cells
- RUNX1-ETO disrupts global RUNX1 binding and deregulates RUNX1 target genes
- RUNX1-ETO blocks myeloid differentiation by rapidly downregulating *SPI1* and *CEBPA*
- The impact of RUNX1-ETO induction is cell type specific

Authors

Monica Nafria, Peter Keane, Elizabeth S. Ng, Edouard G. Stanley, Andrew G. Elefanty, Constanze Bonifer

Correspondence

andrew.elefanty@mcri.edu.au (A.G.E.),
c.bonifer@bham.ac.uk (C.B.)

In Brief

This study uses human-embryonic-stem-cell-derived definitive human blood progenitors to gain insight into the earliest events following leukemic oncogene expression. Nafria et al. show that induction of RUNX1-ETO expression rapidly reprograms chromatin, disrupts global RUNX1 binding, and arrests growth and/or differentiation specifically in early myeloid progenitor cells.

Nafria et al., 2020, Cell Reports *31*, 107691
May 26, 2020 © 2020 The Author(s).
https://doi.org/10.1016/j.celrep.2020.107691

CellPress

Cell Reports

CellPress
OPEN ACCESS

Article

Expression of RUNX1-ETO Rapidly Alters the Chromatin Landscape and Growth of Early Human Myeloid Precursor Cells

Monica Nafria,[1,2,3,5] Peter Keane,[1,5] Elizabeth S. Ng,[2] Edouard G. Stanley,[2,3,4] Andrew G. Elefanty,[2,3,4,*] and Constanze Bonifer[1,6,*]

[1]Institute for Cancer and Genomic Sciences, College of Medicine and Dentistry, University of Birmingham, Birmingham B15 2TT, UK
[2]Murdoch Children's Research Institute, The Royal Children's Hospital, Flemington Road, Parkville, VIC 3052, Australia
[3]Department of Paediatrics, Faculty of Medicine, Dentistry and Health Sciences, University of Melbourne, Parkville, VIC 3052, Australia
[4]Department of Anatomy and Developmental Biology, Monash University, Clayton, VIC 3800, Australia
[5]These authors contributed equally
[6]Lead Contact
*Correspondence: andrew.elefanty@mcri.edu.au (A.G.E.), c.bonifer@bham.ac.uk (C.B.)
https://doi.org/10.1016/j.celrep.2020.107691

SUMMARY

Acute myeloid leukemia (AML) is a hematopoietic malignancy caused by recurrent mutations in genes encoding transcriptional, chromatin, and/or signaling regulators. The t(8;21) translocation generates the aberrant transcription factor RUNX1-ETO (RUNX1-RUNX1T1), which by itself is insufficient to cause disease. t(8;21) AML patients show extensive chromatin reprogramming and have acquired additional mutations. Therefore, the genomic and developmental effects directly and solely attributable to RUNX1-ETO expression are unclear. To address this, we employ a human embryonic stem cell differentiation system capable of forming definitive myeloid progenitor cells to express *RUNX1-ETO* in an inducible fashion. Induction of RUNX1-ETO causes extensive chromatin reprogramming by interfering with RUNX1 binding, blocks differentiation, and arrests cellular growth, whereby growth arrest is reversible following RUNX1-ETO removal. Single-cell gene expression analyses show that RUNX1-ETO induction alters the differentiation of early myeloid progenitors, but not of other progenitor types, indicating that oncoprotein-mediated transcriptional reprogramming is highly target cell specific.

INTRODUCTION

Hematopoietic development and differentiation are regulated through a hierarchical network of DNA-sequence-specific transcription factors. Genetic alterations affecting such regulators impair the balance of interactions within their corresponding transcriptional network, leading to a disturbance of differentiation and enhanced self-renewal. Acute myeloid leukemia (AML) is a heterogeneous disease marked by proliferation of neoplastic cells with impaired myeloid differentiation. The t(8;21) translocation, accounting for approximately 10% of all AML, fuses the DNA binding domain of the hematopoietic master regulator RUNX1 to almost the entire ETO protein (Miyoshi et al., 1991). The resulting RUNX1-ETO fusion protein phenotypically functions as a dominant-negative version of RUNX1 by blocking hematopoietic development both *in vivo* and *in vitro* (Regha et al., 2015; Yergeau et al., 1997). It recruits histone deacetylase complexes to RUNX1 binding sites through its ETO moiety, resulting in repression of genes that regulate hematopoietic differentiation (Lutterbach et al., 1998; Regha et al., 2015). Experiments depleting RUNX1-ETO in established AML cells have shown that it is required to maintain leukemic growth (Ptasinska et al., 2012) but have also demonstrated that RUNX1-ETO-regulated gene expression is complex, with multiple genes being up- and downregulated after knockdown (Ptasinska et al., 2014, 2019), indicating that the entire transcriptional network of such cells is rewired in the presence of the fusion protein.

The t(8;21) translocation can occur early during development and has been detected *in utero* (Wiemels et al., 2002), indicating that its presence does not interfere with general hematopoietic differentiation in human embryos after formation of progenitor cells. Moreover, t(8;21) patients in remission can harbor preleukemic stem cells carrying the translocation but lacking secondary mutations, which may serve as a reservoir for relapse (Miyamoto et al., 2000; Shima et al., 2014). These findings agree with the findings of experiments modeling the disease in mice, demonstrating that RUNX1-ETO alone is not sufficient to cause AML (Higuchi et al., 2002; Yuan et al., 2001). Given that leukemia development requires the acquisition of multiple genetic aberrations, the study of primary cells from patient leukemic samples does not allow easy discrimination of the impact of RUNX1-ETO alone on the gene regulatory network of normal blood progenitor cells. Several studies examined the development of AML

This is an open access article under the CC BY license (http://creativecommons.org/licenses/by/4.0/).

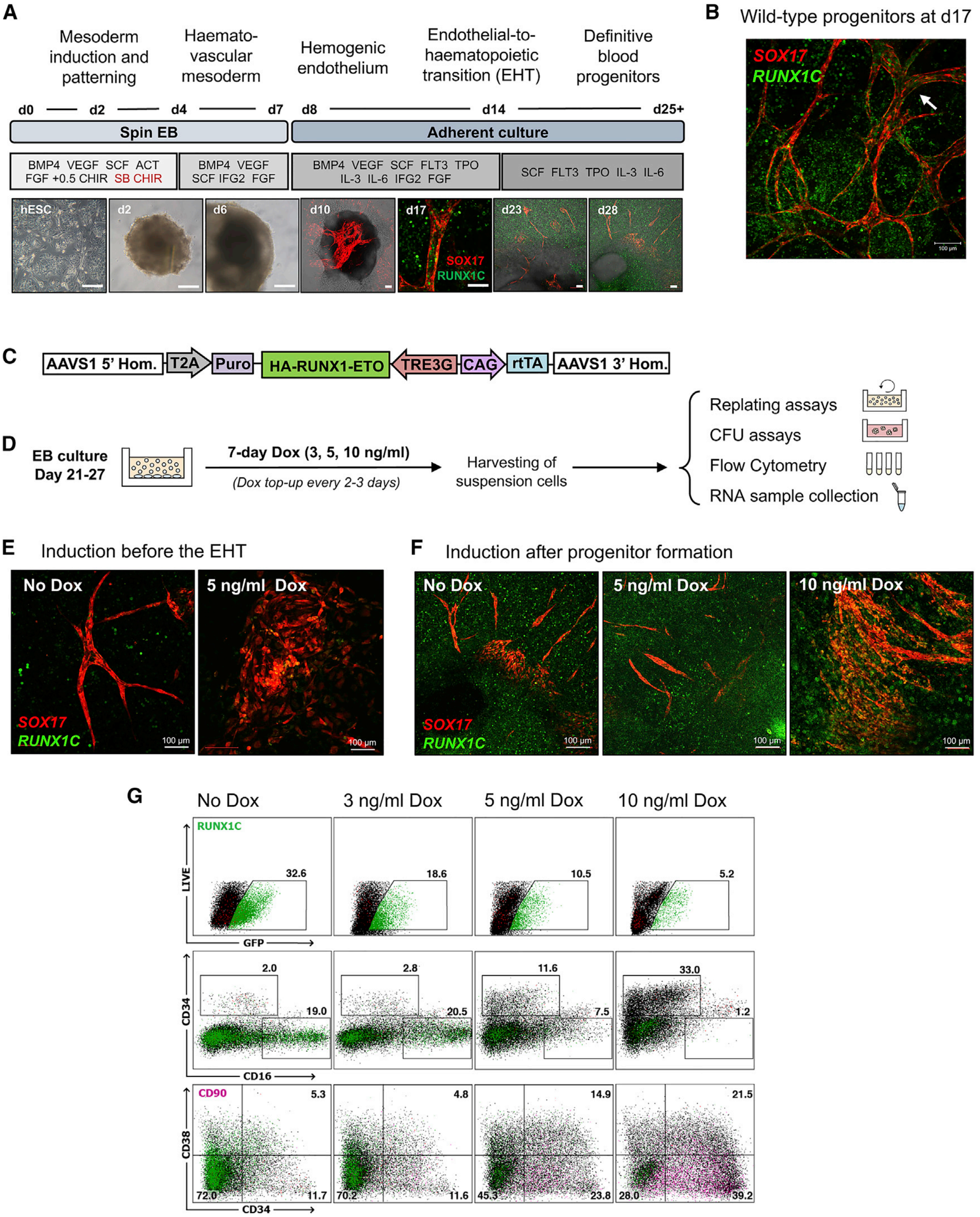

Figure 1. Expression of *RUNX1-ETO* Leads to a Differentiation Arrest of Human Early Hematopoietic Progenitor Cells

(A) Protocol for *in vitro* human definitive hematopoietic differentiation as spin embryoid bodies (EBs). Developmental stages, time course, and growth factors used are indicated. Epi-fluorescence images (human embryonic stem cells [hESCs], days 2 and 6) and confocal images (days 10–28) representative of each stage are shown. A pulse of SB431542 and CHIR (red) was included from days 2–4. hESCs are shown ~50% confluency on a feeder layer. EBs (days 2 and 6) appear as opaque round structures and are surrounded by adherent stroma, endothelium, and blood cells from day 10. Fluorescence and bright-field channels are merged in images corresponding to days 10, 13, and 28. Scale bar: 100 μm. SOX17 (mCHERRY, red) expression marks vascular structures, and RUNX1C (GFP, green) marks hematopoietic progenitors.

(B) Confocal image of a differentiation culture at day 17 showing RUNX1C+ progenitors being generated from SOX17+ vascular structures of endothelial cells, resembling embryonic AGM hematopoiesis. SOX17 (CHERRY, red), RUNX1C (GFP, green). The arrow points to groups of progenitor cells resembling the embryonic intra-aortic hematopoietic cell clusters. Scale bar: 100 μm.

(legend continued on next page)

using inducible RUNX1-ETO expression in mice or constitutive expression in human cells *in vitro*. However,AML development was slow (Cabezas-Wallscheid et al., 2013), the proliferation of cells in the culture dish implied the requirement for a selection step (Mandoli et al., 2016; Mulloy et al., 2002, 2003), RUNX1-ETO was induced at higher levels than those seen in patients (Mulloy et al., 2002, 2003), and assays were performed in established cell lines harboring additional mutations (Martens et al., 2012; Ptasinska et al., 2012, 2014) or in *in vitro*-differentiated hematopoietic cells resembling yolk-sac-like progenitors that are unlikely to represent the proper target cell types (Mandoli et al., 2016; Tijchon et al., 2019). These caveats hinder the understanding of the earliest, unperturbed chromatin reprogramming events occurring in a human setting after RUNX1-ETO induction. To circumvent these limitations, we genetically altered human pluripotent stem cells to activate *RUNX1-ETO* in response to doxycycline (Dox) and used an *in vitro* system of hematopoietic differentiation that biases cultures toward definitive multipotent hematopoietic progenitor cells (Ng et al., 2016).

Our experiments showed that high levels of RUNX1-ETO had a detrimental effect on hematopoiesis. However, levels of *RUNX1-ETO* expression that matched those of endogenous *RUNX1* in immature clonogenic blood progenitors were compatible with cellular survival. Within 24 h of *RUNX1-ETO* induction, cells became quiescent and downregulated hematopoietic differentiation, cell-cycle, and DNA repair genes but upregulated mitogen-activated protein kinase (MAPK) and vascular endothelial growth factor (VEGF) signaling pathway genes. In contrast to uninduced cells, these cells could survive for months *in vitro* without proliferating. Strikingly, following the removal of Dox and the cessation of *RUNX1-ETO*, transcription these immature, quiescent *RUNX1-ETO*-expressing cells revealed an enhanced clonogenic capacity and regained the ability to proliferate and differentiate. Chromatin immunoprecipitation (ChIP) and chromatin accessibility assays showed that RUNX1-ETO binding led to widespread loss of chromatin accessibility at RUNX1 binding sites and substantially altered the RUNX1-controlled transcriptional program. Single-cell RNA sequencing (scRNA-seq) experiments demonstrated that RUNX1-ETO induction exerted its main impact on an early myeloid precursor population, by downregulating the myeloid master regulator PU.1, but had little effect at later stages or different lineages. Our study sheds light on the earliest events occurring after RUNX1-ETO expression in a human primary cell setting and demonstrates a dissociation between block in differentiation and cell proliferation, considered the two hallmarks of leukemia.

RESULTS

Expression of *RUNX1-ETO* Leads to Reversible Differentiation and Growth Arrest of Human Early Hematopoietic Progenitor Cells

To analyze the effects of RUNX1-ETO induction in defined cell types, we generated inducible RUNX1-ETO human embryonic stem cell (ESC) lines. The parental line used was a previously generated human H9 ESC dual reporter cell line (denoted SOX17$^{mCHERRY/w}$RUNX1C$^{GFP/w}$) carrying an *mCHERRY* gene in the *SOX17* locus, marking arterial endothelium (Clarke et al., 2013), and a *GFP* gene in the *RUNX1* locus, resulting in expression of GFP from the distal promoter (*RUNX1C*) and hence marking hematopoietic stem cells (HSCs) and progenitor cells (Ng et al., 2016; Figure S1A). *RUNX1C* is the dominant *RUNX1* isoform in fetal liver blood progenitors (Sroczynska et al., 2009). In contrast to *RUNX1B*, *RUNX1C* expression is restricted to hematopoietic cells and defines the subset of CD34+ cells with clonogenic and bone marrow homing activity (Ng et al., 2016). This strategy allowed us to track the progression of *in vitro* cell differentiation (Figures 1A and 1B; Figure S1B), thus facilitating the distinction between endothelial and hematopoietic cells. It also allowed us to monitor the endothelial-to-hematopoietic transition (EHT), the process by which hematopoietic progenitor cells form and detach from the endothelium, which is accompanied by a switch in *RUNX1* promoter usage (Bertrand et al., 2010; Boisset et al., 2010; de Bruijn et al., 2002; Jaffredo et al., 1998; Kissa and Herbomel, 2010; Lam et al., 2010; North et al., 2002). We derived definitive hematopoietic progenitors from human ESC lines using a protocol that included SB431542 and CHIR (an ACTIVIN inhibitor and a WNT agonist, respectively) from day 2–4 to pattern cells toward an intra-embryonic, definitive *HOXA*+ fate (Ng et al., 2016; Figure 1A). By differentiating the dual reporter cell line using this protocol, we

(C) Schematic representation of the transgene targeting the AAVS1 locus. Knockin was performed via transcription activator-like effector nuclease (TALEN)-mediated homologous recombination into the AAVS1 locus of the SOX17$^{mCHERRY/w}$ RUNX1C$^{GFP/w}$ hESC line H9. The integrated sequence includes an HA-tagged *RUNX1-ETO* cDNA under control of a tetracycline-inducible expression system (TRE-3G), the reverse tetracycline activator (rtTA) controlled by a chicken β-actin promoter (CAG), and a puromycin resistance gene with an upstream T2A sequence to link its expression to the AAVS1 gene.

(D) Experimental strategy for the evaluation of RUNX1-ETO induced by Dox (0, 3, 5, or 10 ng/mL). Cultures were treated with Dox at different time points once blood progenitors were had been formed (days 21–27). The non-adherent hematopoietic cell fraction was harvested 7 days after induction and used for flow cytometry analysis and CFU and replating assays.

(E) Confocal images of hematopoietic cultures at day 17, showing the disruptive effects on vasculogenesis and blood formation of RUNX1-ETO induction before the EHT, which occurs around day 12 in our cultures. Images are representative of uninduced and induced cultures at day 10 (5 ng/mL Dox for 7 days). SOX17 (CHERRY) and RUNX1C (GFP). Scale bar: 100 μm.

(F) Confocal images of hematopoietic cultures at day 28 showing the effect of RUNX1-ETO on formation of blood progenitors. *RUNX1-ETO* expression at an equivalent level to that of endogenous RUNX1 (5 ng/mL Dox) allows vasculogenesis and blood formation to occur, whereas higher *RUNX1-ETO* expression levels (10 ng/mL Dox) result in the formation of abnormal vascular structures and reduced blood formation. Images are representative of uninduced and induced cultures at day 21 with 5 or 10 ng/mL Dox for 7 days. SOX17 (CHERRY) and RUNX1C (GFP). Scale bar: 100 μm.

(G) *RUNX1-ETO*-expressing cultures retain markers of immature myeloid progenitors. Flow cytometry analysis of the floating fraction of day 34 hematopoietic progenitors upon 7-day RUNX1-ETO induction (3, 5, or 10 ng/mL Dox at day 27). Results are representative of three biological replicates with comparable results with induction at different time points after the EHT. Accumulated precursor cells are highlighted pink.

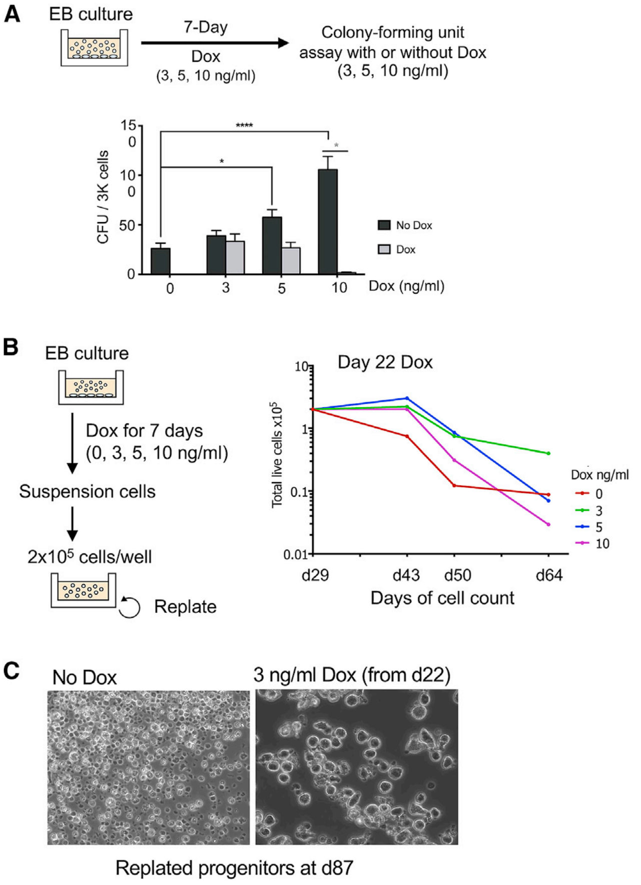

Figure 2. RUNX1-ETO Induction Leads to Increased Survival and a Reversible Growth Arrest

(A) RUNX1-ETO induction causes a reversible block in colony-forming ability. Top: diagram depicting the experimental strategy. EB cultures were treated with 3, 5, or 10 ng/mL Dox for 7 days, and suspension cells were subsequently plated in methylcellulose for CFU assays in the presence or the absence of Dox. Below: CFU assay of day 31 progenitors from treated EB cultures (at day 24 for 7 days), plated in methylcellulose in the presence (light gray) or absence (dark gray) of Dox. Data are from three independent biologic replicates using two clones. CFU assays were conducted in triplicate, with 3,000 cells plated per well. Error bars represent the standard error of the mean (SEM). Gray asterisk: multiple t test, statistical significance determined using the Holm-Sidak method, with alpha = 0.05. Each row was analyzed individually, without assuming a consistent SD. Black asterisk: two-way ANOVA, statistical significance determined using Dunnett's multiple comparison test.

(B) Induction of RUNX1-ETO at low levels (3 and 5 ng/mL Dox) enhances the survival of a subset of progenitor cells compared with uninduced cells, without increasing proliferative capacity. Left: schematic of the replating assays. EB cultures were treated at different stages of hematopoietic differentiation with 0, 3, 5, or 10 ng/mL Dox for 7 days. Floating progenitors were harvested and plated on Matrigel-coated wells at 2×10^5 cells/well in the corresponding Dox concentration and were serially passaged each week. Right: cell count of live cells $\times 10^5$ during replating assays of hematopoietic progenitors from day 29 cultures previously treated with Dox for 7 days (from day 22), showing one representative of three biological replicates. Cell growth was measured at the indicated times.

(C) RUNX1-ETO expressed at low levels allows survival of cells until day 87. Bright-field images of hematopoietic progenitors from replating assays at day 87 of differentiation that are uninduced (left) or treated with 3 ng/mL Dox from day 22 onward (right). Images are taken using the same magnification.

were able to visualize RUNX1C+ progenitors emerging from SOX17+ hemogenic endothelium, forming cell clusters that resembled progenitor formation within the 5th week of human embryonic development in the aorta-gonad-mesonephros (AGM) (Ng et al., 2016; Figure 1B; Figure S1B).

We next generated cell lines carrying an N-terminal hemagglutinin (HA)-tagged *RUNX1-ETO* cDNA, expressed in a Dox-inducible manner and targeted to the safe harbor AAVS1 locus (Figure 1C). Induction of HA-*RUNX1-ETO* occurred homogeneously within the cell population (Figures S1C and S1D). We first determined the Dox concentration required to induce *RUNX1-ETO* expression in hematopoietic progenitors to levels that mimicked the balanced ratio of *RUNX1-ETO*:*RUNX1* expression observed in AML patients (Figure S1E), suggesting 3–5 ng/mL Dox as the most appropriate concentrations. Induction with 5 ng/mL Dox yielded RUNX1-ETO protein levels equivalent to those of endogenous RUNX1 (Figure S1F). Higher Dox concentrations (100–500 ng/mL) that further increased *RUNX1-ETO* levels abrogated blood formation (data not shown). To examine the dose responsiveness of hematopoiesis to *RUNX1-ETO* expression, we induced differentiating cells with 3, 5, or 10 ng/mL Dox for 7 days (Figure 1D). Dox induction before the EHT resulted in severe disorganization of the SOX17+ vasculature and an overall reduction of blood cells that also appeared phenotypically abnormal (Figure 1E; Figure S1G). Abnormal progenitors either lacked *RUNX1C* (GFP) expression, failed to downregulate *SOX17* (CHERRY+), or co-expressed *SOX17* and *RUNX1C* (CHERRY+ and GFP+). However, induction after the EHT allowed the generation of phenotypically normal blood cells, with only 10 ng/mL Dox reducing blood cell numbers (Figure 1G). Expression of *RUNX1-ETO* affected the nature of the hematopoietic cells present in our cultures, causing a Dox-dependent decrease of RUNX1C+ and CD16+ myeloid cells and an increase of CD34+CD38−CD90+ populations resembling immature blood progenitors (Figure 1G).

RUNX1-ETO induction reduced colony-forming ability in a dose-dependent manner in colony-forming unit (CFU) assays in Dox-containing methylcellulose medium (Figure 2A; Figure S1H, left panel). However, CFU activity was restored once Dox was removed from the methylcellulose (Figure 2A; Figure S1H, right panel), indicating reversibility of the RUNX1-ETO-dependent proliferation block. The presence or absence of Dox did not affect colony size or morphology, suggesting that the clonogenic cells differentiated normally following removal of Dox (data not shown). Moreover, long-term culture of hematopoietic cells in the presence of low levels of RUNX1-ETO (3–5 ng/mL Dox) prolonged cell survival without resulting in cell proliferation (Figures 2B and 2C; Figures S1I and S1J). In addition, the presence of RUNX1-ETO caused a profound decrease in DNA-synthesis activity because of an arrest in the G1 phase of the cell cycle, as measured by bromodeoxyuridine (BrdU) incorporation, without an increase in cell death (Figure S1K).

In summary, our experiments show that the expression of balanced levels of *RUNX1-ETO* in developing human blood progenitor cells leads to a reversible block in differentiation with growth arrest and prolongation of cell survival.

CellPress
OPEN ACCESS

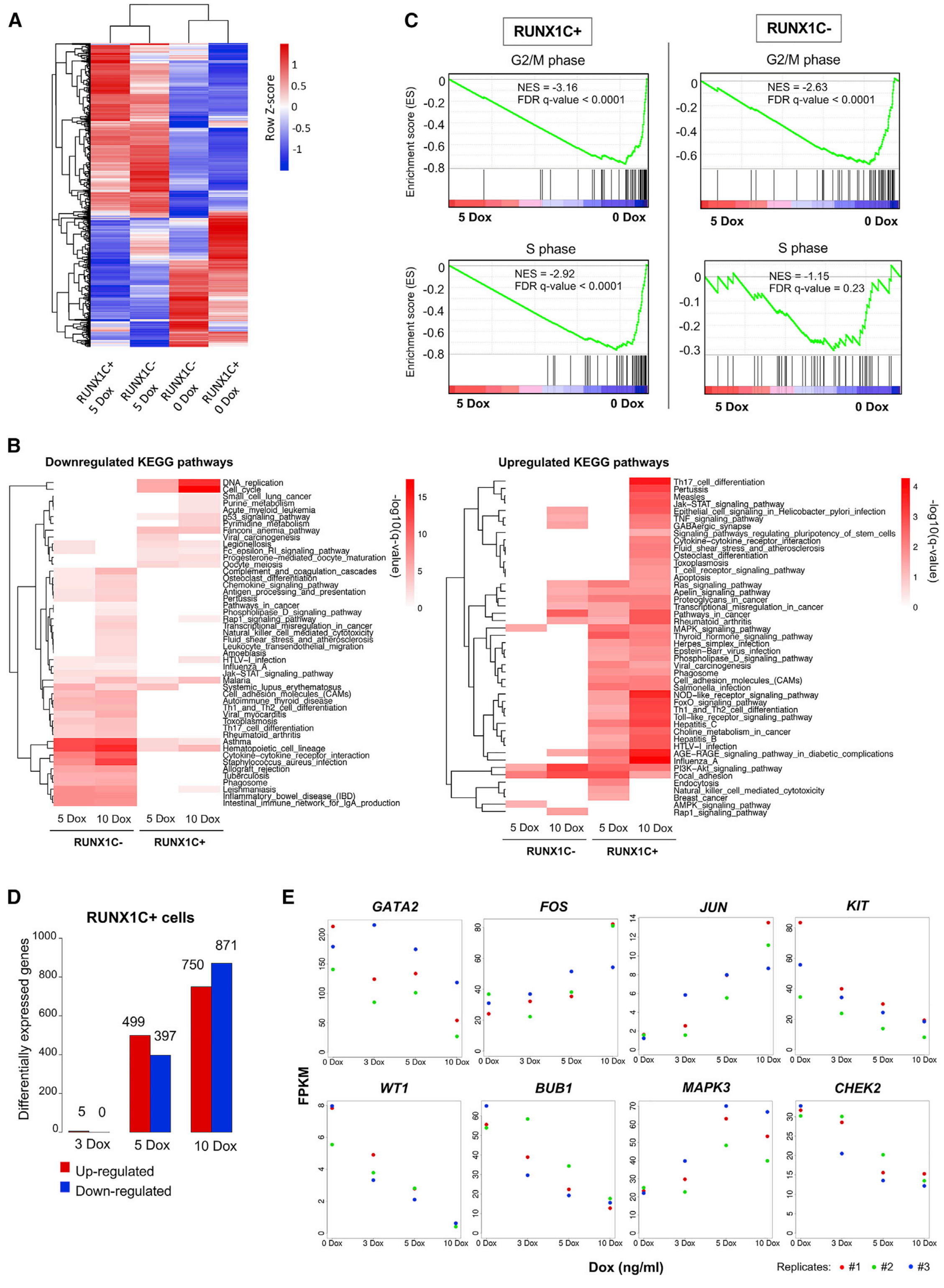

Figure 3. RUNX1-ETO Induction Leads to Cell-Type and Dose-Dependent Changes in Gene Expression

(A) Clustering of gene expression data for sorted populations of RUNX1C− and RUNX1C+ (CD45+CD34+) cells, both wild-type and after 24 h of RUNX1-ETO induction using 5 ng/mL Dox. The figure includes all genes that showed up-/downregulation after RUNX1-ETO induction in either the RUNX1C+ or the RUNX1C− cell populations.

(legend continued on next page)

RUNX1-ETO Induction Leads to Cell-Type and Dose-Dependent Changes in Gene Expression

We previously demonstrated that during mouse *in vitro* hematopoietic specification, RUNX1-ETO reshapes the gene regulatory network in a developmental stage-specific manner and that the outcome depends on the cell type in which the oncogenic event occurs (Regha et al., 2015). To examine the molecular basis of this finding in human cells, we examined genes induced by RUNX1-ETO in two progenitor cell populations (SOX17−CD45+CD34+RUNX1C+$^{(GFP+)}$ and SOX17−CD45+CD34+RUNX1C−$^{(GFP-)}$), isolated from day 22 differentiating cultures, with or without prior induction of RUNX1-ETO with 24 h of Dox treatment (Figure S2A, Data S1). Both populations were composed of CD34+CD45+ progenitors, but the molecular distinction between RUNX1C+ and RUNX1C− populations under uninduced conditions was not known. Analysis of differential gene expression between uninduced RUNX1C+ and RUNX1C− cells showed more than 1,100 differentially expressed genes, demonstrating an intrinsically different nature of the two cell populations (Figure S2B). Expression levels of *RUNX1* and *SPI1* were similar in both populations, but RUNX1C− cells expressed high levels of monocyte-specific genes such as *IRF8*, *CSF1R*, and *CD14*, indicating the presence of maturing myelomonocytic cells (Data S2). RUNX1C+ cells expressed higher levels of *MYB*, *GATA2*, and *GFI1*, as well as the erythroid regulators *GATA1* and *KLF1*. After induction of RUNX1-ETO with 5 ng/mL Dox, both RUNX1C− and RUNX1C+ cell populations up- and downregulated similar numbers of genes (Figure S2C). However, the actual RUNX1-ETO-responsive gene expression program was different (Figure 3A; Data S3). In accordance, up- and downregulated Kyoto Encyclopedia of Genes and Genomes (KEGG) pathways in gene sets responding to 5 or 10 ng/mL Dox induction differed as well (Figure 3B; Data S4). RUNX1C+ cells downregulated cell-cycle and DNA replication genes (such as *BRCA1*, *BUB1B*, and *RNASEH2B*) and upregulated a large number of signaling genes (such as *MAPK3* and *JUN*), whereas RUNX1C− cells downregulated genes belonging to hematopoietic lineage pathways (such as *CEBPA*, *IL4*, and *KIT*) and upregulated only a subset of the genes upregulated in the RUNX1C+ population. In addition, upon treatment with 5 ng/mL Dox, RUNX1C+ cells showed downregulation of genes related to both the G2/M and S phases of the cell cycle (Figure 3C, right panels), agreeing with the observed cell-cycle arrest upon RUNX1-ETO induction (Figure S1J). In contrast, RUNX1C− cells did not downregulate S-phase genes upon induction, suggesting that these cells were still able to replicate (Figure 3C, left panels). The gene expression response to RUNX1-ETO induction in RUNX1C+ cells was highly dose dependent (Figure 3D; Figure S2D). Furthermore, RUNX1-ETO induction yielded highly heterogeneous changes in gene expression, with distinct subsets of genes responding differently to the oncogene dosage (Figure S2E). For example, expression of the stem cell regulator *GATA2* and the *WT1* gene decreased, along with cell-cycle genes (*BUB1* and *CHEK2*) and the growth factor receptor gene *KIT* (Figure 3E). In contrast, genes involved in signaling pathways (*MAPK3*) and immediate-early response genes (*FOS* and *JUN*) were upregulated (Figure 3E). These gene expression data were concordant with the observed cell-cycle arrest and demonstrate that RUNX1-ETO affects distinct cell types differently, suggesting that RUNX1-ETO induction in the appropriate cell type is crucial for understanding how it reprograms the epigenome of myeloid cells.

RUNX1-ETO Induction Causes Extensive Global Chromatin Reorganization and Disrupts the Binding of RUNX1 at Distal Elements

To understand the RUNX1-ETO-mediated chromatin reprogramming and changes in transcription factor binding in RUNX1C+ cells, we next analyzed open chromatin regions, using the assay for transposase-accessible chromatin with high-throughput sequencing (ATAC-seq), and protein binding, by performing ChIP experiments (Figure 4A, Data S1). Induction of RUNX1-ETO shifted the accessible chromatin landscape (Figure 4B, ATAC-seq); an example for this is the *SPI1* (PU.1) locus that is shown in Figure 4C. A large number of accessible sites were lost (5,419) and gained (4,112) within 24 h of Dox induction. Lost sites were associated with downregulated gene expression (Figure 4B, gene expression) and were enriched in binding motifs for important members of hematopoietic transcription factor families, such as PU.1 (but not ETS), as well as GATA, RUNX, and C/EBP family members (Figures 4B, motif density plots, and 4D). These results were confirmed by chromatin immunoprecipitation sequencing (ChIP-seq) experiments showing loss of RUNX1 binding across all RUNX1-ETO bound sites, as well as a reduction of the active histone marks histone H3 lysine 27 acetylation (H3K27ac) and histone H3 lysine 4 trimethylation (H3K4me3) (Figures 4B and 4E). These losses were most pronounced on distal elements (Figures S3C and S3D), whereas promoters were less affected (Figures S3A and S3B). RUNX1-ETO has been shown to associate with several transcription factors, forming a complex containing LMO2, LDB1, and E-Box binding factors, such as LYL1 and ETS family members ERG/FLI1, all of which were shown to be important for chromatin binding and leukemogenesis (Martens et al., 2012; Sun et al., 2013). To test how the binding of these factors

(B) Heatmap after KEGG pathway analysis depicting clustering of differentially expressed genes associated with KEGG terms upon RUNX1-ETO induction (5 and 10 ng/mL Dox) in sorted populations of both RUNX1C− and RUNX1C+ cells. Red intensity reflects the enrichment significance of the terms in −log10 (q value).

(C) Gene set enrichment analysis (GSEA) correlating expression of genes differentially regulated during the cell cycle (G2/M in top panels and S phase in bottom panels) between induced (5 ng/mL Dox for 24 h) and uninduced conditions in both RUNX1C+ and RUNX1C− cell populations. ES, enrichment score; NES, normalized enrichment score; FDR, false discovery rate.

(D) Bar graph depicting differentially expressed genes between uninduced and Dox-treated (3, 5, and 10 ng/mL Dox) sorted populations of CD45+CD34+RUNX1C+ cells.

(E) Examples of individual genes differentially regulated in CD34+RUNX1C+ progenitors in response to RUNX1-ETO induction (3, 5, and 10 ng/mL Dox) for 24 h. n = 3. Each colored dot represents a distinct biological replicate.

CellPress
OPEN ACCESS

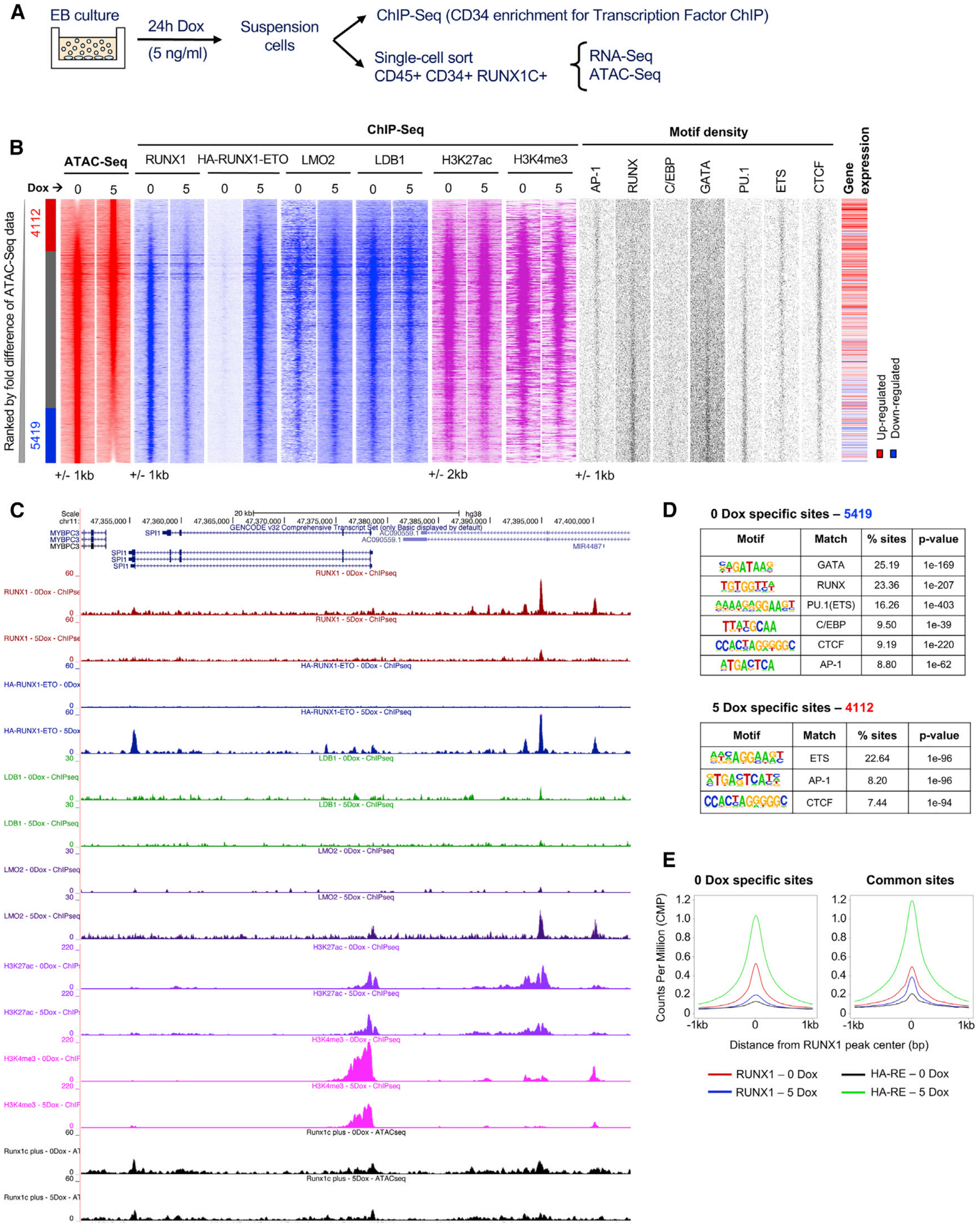

Figure 4. RUNX1-ETO Induction Causes Extensive Global Chromatin Reorganization and Blocks the Binding of RUNX1

(A) Sorting strategy and downstream analyses after 24-h induction of RUNX1-ETO with 5 ng/mL Dox.

(B) Heatmaps depicting accessible chromatin regions ranked by the fold difference between 0 and 5 ng/mL Dox RUNX1C+-treated samples. ATAC-seq peaks were considered sample specific when displaying a greater than 2-fold enrichment compared with the other sample. Sample-specific sites and number of peaks are indicated alongside: red, 5-Dox specific; blue, 0-Dox specific; gray, shared peaks. ChIP-seq enrichment for RUNX1, HA-RUNX1-ETO, LMO2, LDB1, H3K27ac, and H3K4me3 in each sample; motif density plots; and gene expression at these sites are ranked along the same coordinates as the ATAC-seq peaks.

(legend continued on next page)

responded to RUNX1-ETO induction, we measured their binding to both promoter and distal regulatory regions in the presence or absence of RUNX1-ETO using ChIP (Figures 4B and 4C; Figures S3A and S3C). The binding of RUNX1-ETO was accompanied by an increase in LMO2 binding, whereas the binding of LDB1 appeared to follow the loss of RUNX1. This result is in concordance with LDB1 and RUNX1-ETO knockdown experiments that showed that the reduction of LDB1 binding did not influence the binding of RUNX1-ETO but followed the *de novo* binding of RUNX1 and the establishment of new *cis*-element interactions after RUNX1-ETO depletion (Ptasinska et al., 2019). Loss of RUNX1 binding at specific sites was directly correlated to RUNX1-ETO expression levels (Figure 5A), with some genomic sites presenting total abrogation of RUNX1 binding upon induction of RUNX1-ETO with 10 ng/mL Dox, as exemplified in the *RASSF5* locus (Figure 5B), thus demonstrating that the two factors are in direct competition. This idea is supported by most genes that were bound by both factors showing loss of RUNX1 binding after RUNX1-ETO induction (Figure S3E, circles intersection, purple). Around 40% of both upregulated and downregulated genes upon induction were RUNX1-ETO target genes (Figure S3F). Moreover, most differentially expressed RUNX1 targets that lost RUNX1 binding upon induction were RUNX1-ETO target genes, as well (Data S5), with a smaller proportion of non-targets (Figure S3F, light colors). Altogether, our data demonstrate that induction of RUNX1-ETO strongly interferes with the RUNX1-driven gene expression program, mostly by direct competition but also by indirect means.

Human ESC-derived multipotent progenitor cells show a transcriptome pattern similar to those of the first pre-HSCs developing from the human AGM (Ng et al., 2016). However, changes in the chromatin structure often precede the onset of transcription (Bonifer and Cockerill, 2017). We therefore wished to determine to what extent the chromatin landscape in our human ESC-derived progenitors resembled that of normal blood stem and progenitor cells. To this end, we compared our ATAC-seq data derived from uninduced and induced cells to those generated from highly purified human hematopoietic precursor populations, as well as monocytic cells (Corces et al., 2016; Figure 5C). This analysis shows a strong resemblance of the overall ATAC-seq pattern of human ESC-derived RUNX1C+ progenitors to HSC and multi-potent progenitor (MPP) populations, but not to monocytes. RUNX1-ETO induction shifted this pattern, resulting in loss of open chromatin regions specific for early progenitors and appearance of new accessible sites. In human ESC-derived progenitors, RUNX1-ETO induction deregulated a set of genes similar to that found in t(8;21) AML patients when compared with normal CD34+ stem/progenitor cells (Figure 5D). This result argues that the initial RUNX1-ETO mutation accounts for a large portion of the altered transcriptional network in leukemia patient samples.

RUNX1-ETO Acts on Early Myeloid Cells

To identify the cells most responsive to RUNX1-ETO induction, we performed scRNA-seq experiments, comparing purified CD45+CD34+RUNX1C+ cells with or without 24-h treatment with 5 ng/mL Dox, assigning cell populations based on expression of known lineage marker genes (Figure 6A; Figures S4A–S4C; Data S6). The clustering analysis in Figure 6B shows that the uninduced population consisted of early erythroid precursors, eosinophils, together with maturing erythroid and myeloid cells, and a population of less differentiated cells resembling stem and different types of myeloid progenitor cells, as described by Drissen et al. (2019; Figure 6B, left panel). Overnight induction of RUNX1-ETO led to the emergence of an enriched population (Figure 6B, right panel, green; Figure S4D), herein referred as the 5-Dox-enriched population. Analysis of cell-cycle-regulated genes within this population showed a strong arrest in the G1 phase of the cell cycle (Figure 6C). Some degree of cell-cycle arrest was also observed in the stem/progenitor, eosinophil, and guanosine monophosphate (GMP)-like populations (orange/purple/dark blue), but not in the erythroid populations (pink/red). A similar result was seen when we measured the number of genes responding to induction (Figure S4E), with the 5-Dox-enriched population (green) presenting the highest number of changing genes, followed by the eosinophil progenitor population (orange) and lastly the erythroid lineage cells (pink and red), which showed only a few responsive genes. We next projected the expression of important hematopoietic regulator genes on the different cell clusters (Figure 6D). This analysis demonstrated again the differential response to RUNX1-ETO induction between the cell clusters. The quantification of expression of individual genes (Figure S5) showed that the most pronounced response was the loss of *SPI1* (PU.1) expression in the 5-Dox-enriched population, as well as strong upregulation of expression of the *SOX4* transcription factor gene. We also observed a reduction of *GATA2*, *CEBPA*, and *GFI1B* expression. Cells in the 5-Dox-enriched population expressed the chemokine gene *CCL5*, which has roles in proliferation, metastasis, and creating an immunosuppressive environment. As shown earlier, transcription factors relevant for erythroid development and erythroid lineage genes were largely unaffected, as were other genes such as *MEIS1*. Gene expression and KEGG pathway analysis of up- and downregulated genes in the 5-Dox-enriched population are consistent with the results obtained from the bulk RNA-seq data (Figures S6A and S6B; Data S7). These results show that single-cell analysis of RUNX1-ETO transcriptional dysregulation yielded results similar to the differential gene expression observed in the bulk progenitor population upon 5 ng/mL Dox treatment, confirming the RUNX1-ETO-dependent downregulation of cell cycle, replication, and interestingly, spliceosome and ribosomal genes.

(C) Genome browser screenshot at the *SPI1* gene locus depicting RUNX1, HA-RUNX1-ETO, LMO2, LDB1, H3K27ac, and H3K4me3 ChIP-seq and ATAC-seq tracks in uninduced and induced (5 ng/mL Dox for 24 h) samples.

(D) Motif enrichment analysis in the 0- and 5-Dox-specific peaks.

(E) Average profiles for RUNX1 and RUNX1-ETO ChIP-seq data centered on RUNX1 binding peaks (±1,000 bp from peak center) in the 0- and 5-Dox-specific peaks.

CellPress
OPEN ACCESS

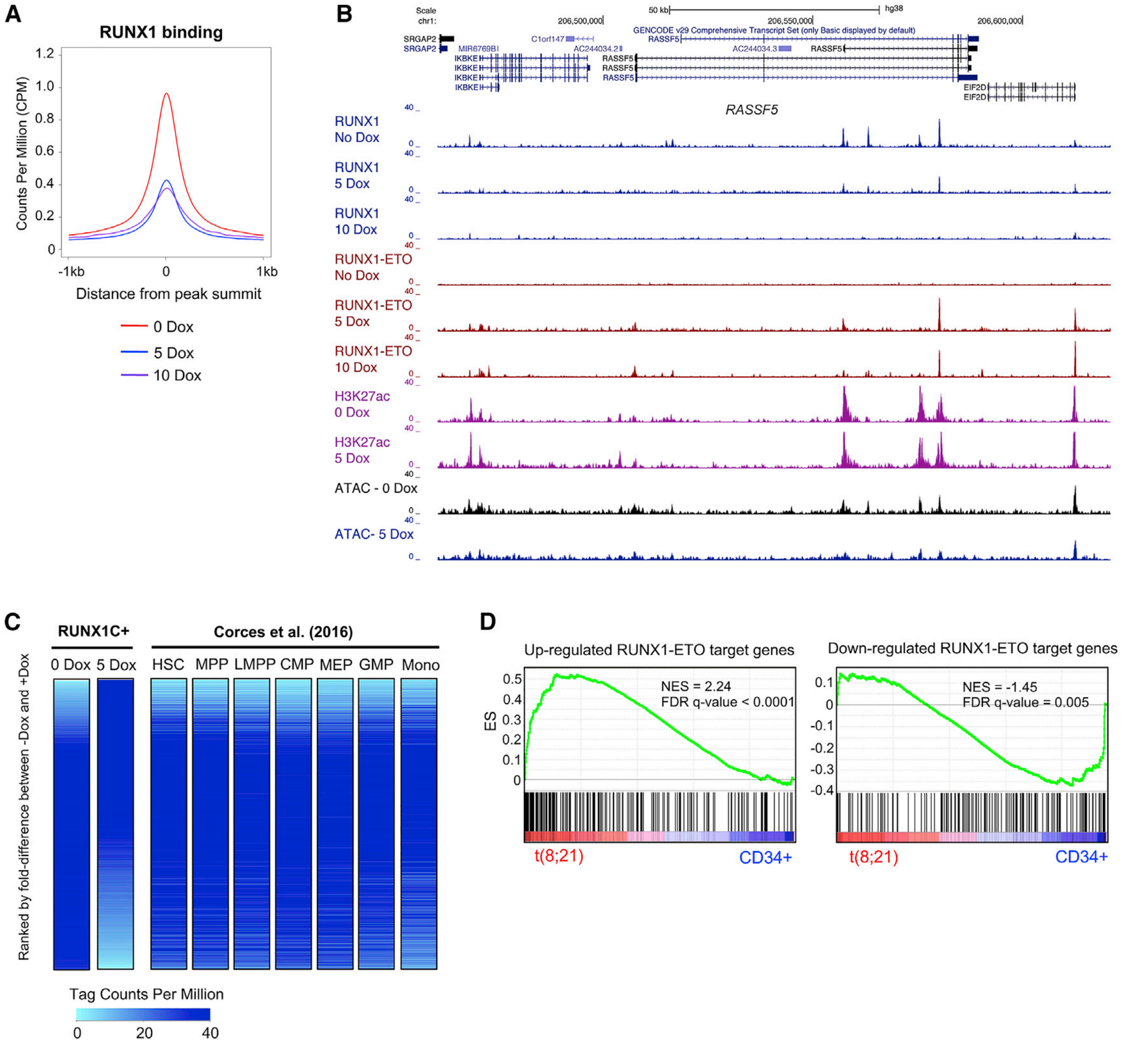

Figure 5. Analysis of the Interplay between RUNX1 and RUNX1-ETO

(A) Average profiles for RUNX1 ChIP-seq tag counts centered on all RUNX1 binding peaks (±1,000 bp from peak center) in the 0, 5, and 10 ng/mL Dox-treated samples.

(B) Genome browser screenshot at the *RASSF5* gene locus depicting RUNX1, HA-RUNX1-ETO, and H3K27ac ChIP-seq and ATAC-seq tracks for the indicated samples.

(C) Comparison of chromatin accessibility in RUNX1C+ cells (0- and 5-Dox-treated samples) to myeloid progenitor cell types from Corces et al. (2016). Heatmaps show ATAC-seq tag counts ranked by fold difference between 0- and 5-Dox-treated RUNX1C+ samples. ATAC-seq tag counts from distinct myeloid progenitor cell types (Corces et al., 2016) are ranked along the same coordinates as the 0-Dox ATAC-seq peaks. Color intensity reflects tag counts per million, with light blue representing closed chromatin.

(D) RUNX1-ETO-induced hESC-derived progenitors share a t(8;21) AML-specific gene expression profile with t(8;21) AML patients. GSEA correlating upregulated (left panel) and downregulated (right panel) RUNX1-ETO target genes between CD45+CD34+RUNX1C+ cells following 24-h RUNX1-ETO induction (5 ng/mL Dox) and the gene expression profile of the RUNX1-ETO targets in t(8;21) patients.

To gain more insight into the position of the RUNX1-ETO-responsive cell population within the differentiation trajectory, we performed a pseudo-time (nearest neighbor) analysis (Figure 7). Uninduced cells showed a clear distribution of the different populations, with the eosinophil progenitor (orange), the myeloid (blue), and the erythroid progenitor lineages branching off (Figure 7A, left panel). Induction of RUNX1-ETO distorted this differentiation trajectory (Figure 7A, right panel); although the erythroid branch was unaffected, all other arms were disorganized, with cells from different clusters scattered over the trajectory. Our results are consistent with RUNX1-ETO halting differentiation, with cells forming a continuum of mixed differentiation stages rather than a trajectory, which would be in agreement with a blocked cell cycle and inability to properly execute lineage fate decisions=. The same scenario could be seen when the expression of specific genes in the different cell populations was projected on the trajectory (Figure 7B). The expression of genes such as *GATA2* and *SPI1* (PU.1) was scattered

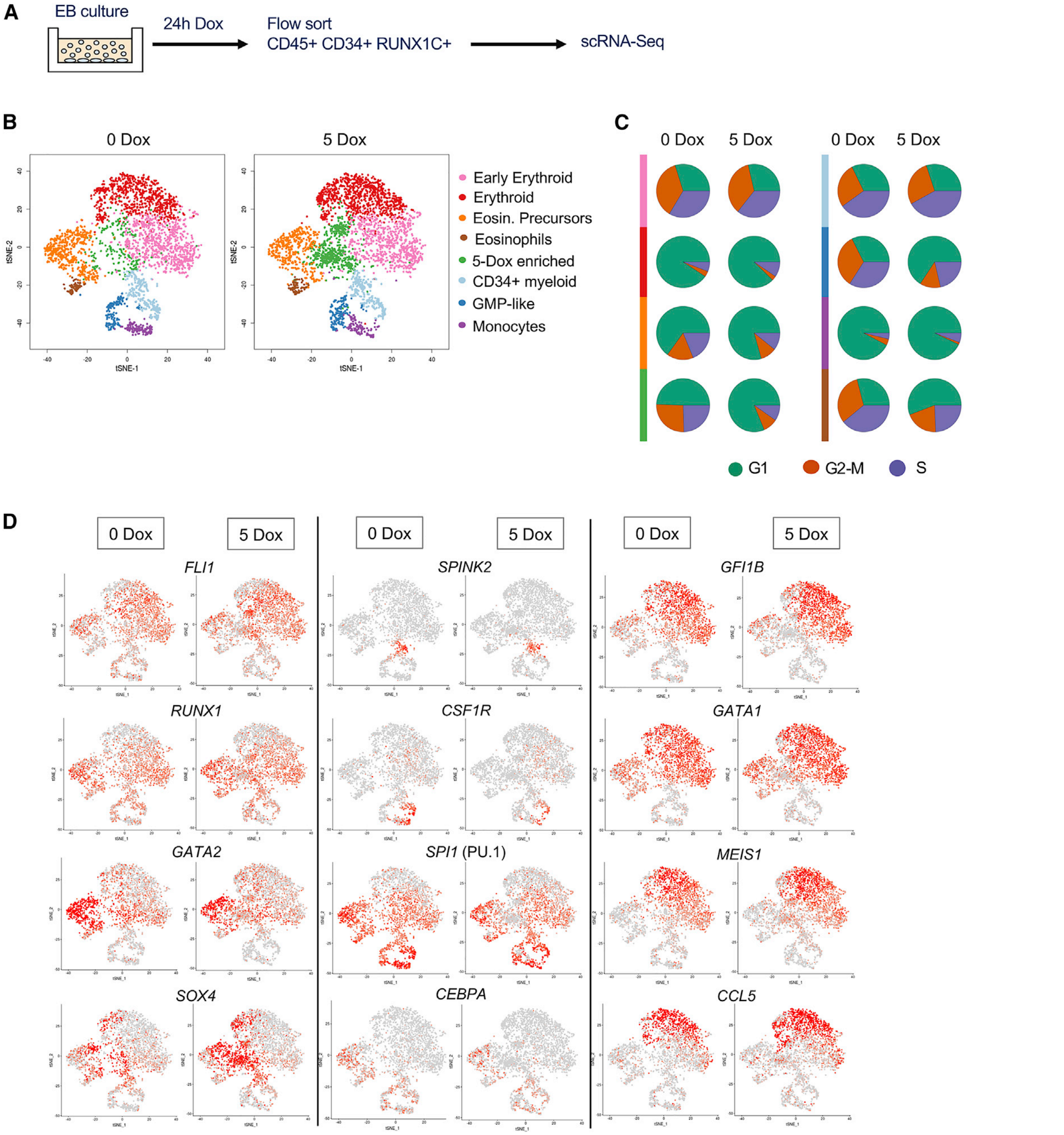

Figure 6. Induction of RUNX1-ETO in the CD45+CD34+RUNX1C+ Population Results in the Emergence of a New Cell Population

(A) Diagram of the sorting strategy for scRNA-seq performed following 24-h induction of RUNX1-ETO (5 ng/mL Dox at day 21).

(B) Two-dimensional t-distributed stochastic neighbor embedding (t-SNE) maps displaying 3,321 (left) and 3,814 (right) sorted populations of CD45+CD34+RUNX1C+ single cells following 0 and 5 ng/mL Dox treatment, respectively. Colors represent the different clusters identified after RaceID analysis.

(C) Pie charts displaying the proportion of cells in each cell-cycle phase (G1, G2-M, and S) within each cell cluster as identified by expression of cell-cycle-regulated genes, such as histone genes.

(D) Expression of individual marker genes projected on the t-SNE maps of both untreated (0 Dox) and treated (5 Dox; 5 ng/mL for 24 h) scRNA populations. Color intensity represents number of transcripts sequenced in log2 of unique molecular identifier (UMI) counts +1.

all over the trajectory after induction (Figure 7B), and their expression was downregulated in eosinophil progenitor (orange) and 5-Dox-enriched (green) populations (Figure 7B; Figure S5). However, the analysis of *SPI1* (PU.1) expression in monocytes (purple) showed a different picture (Figure 7B; Figure S5), because *SPI1* appeared to be less perturbed by RUNX1-ETO induction in this population, suggesting that cells that have passed a certain differentiation state become less sensitive to

CellPress
OPEN ACCESS

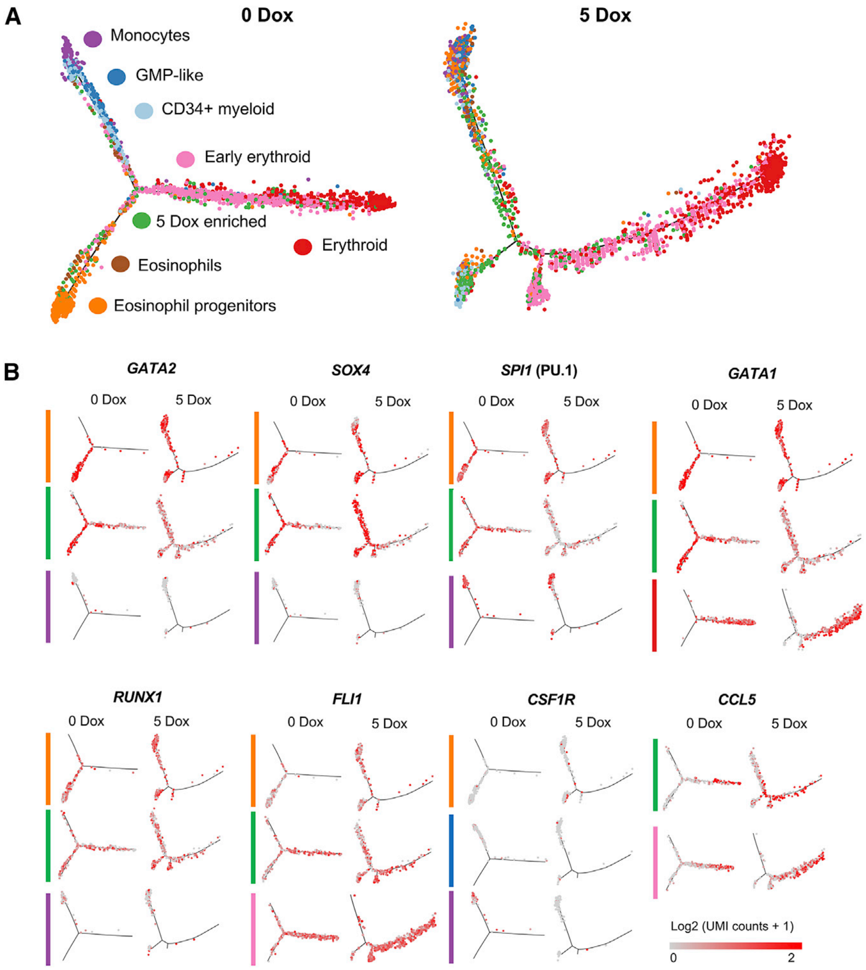

Figure 7. RUNX1-ETO Induction Distorts the Myeloid, but Not the Erythroid, Differentiation Trajectory and Dysregulates Genes Involved in Stem/Progenitor Development

(A) Trajectory analysis using the Monocle algorithm of the sorted cell populations plotted according to each cell cluster.

(B) Expression of individual marker genes projected on the trajectories, plotted according to each cell cluster in (A). Color intensity represents number of transcripts sequenced in log2 of UMI counts +1.

perturbation. This finding could be explained by activation of additional enhancers that do not depend on prior expression of RUNX1 (Leddin et al., 2011). In contrast, *SOX4* upregulation was confined to the eosinophil progenitor and the 5-Dox-induced cell population (Figure S5).

Altogether, our results are consistent with the idea that RUNX1-ETO reprograms an early myeloid cell population, leading to the dysregulation of genes involved in stem/progenitor cell development, followed byarrested differentiation and cellular growth.

DISCUSSION

RUNX1-ETO Induction at Balanced Levels in Human Progenitor Cells Induces Quiescence and Promotes Their Survival

The t(8;21) translocation can be detected *in utero* and *RUNX1-ETO*-expressing clones can be found in post-natal blood samples, suggesting that cells that acquire the mutation might form a pre-leukemic clonal reservoir (Wiemels et al., 2002). Our laboratories have established a protocol for the generation of CD34+RUNX1C+ definitive hematopoietic progenitors arising from *HOXA*+ hemogenic vasculature that resemble cells generated during human intra-embryonic hematopoiesis within the AGM region (Ng et al., 2016). These progenitors present transcriptional profiles, cell surface receptors, and signaling molecules similar to those of cells sorted from human AGM. The work presented here demonstrates that such cells also display an accessible chromatin landscape resembling the HSC/MPP pattern found in adult hematopoietic cells.

In agreement with previous experiments in murine ESCs (Regha et al., 2015), *RUNX1-ETO* expression at high levels in differentiating human ESCs abrogated blood formation, and here we show that it perturbs vasculogenesis, similar to what has been shown in transgenic mice (Yergeau et al., 1997). Expression of *RUNX1-ETO* before the EHT, even at an equivalent level to that of endogenous *RUNX1*, caused substantial disorganization of vascular structures and formation of morphologically abnormal hematopoietic progenitors. However, induction of the same level of RUNX1-ETO after the EHT allowed the formation of progenitor cells but promoted the accumulation of cell populations expressing markers of immature blood progenitors with a CD34+CD38−CD90+ phenotype. This CD34+CD38−CD90+CD45+RUNX1+ signature in our *RUNX1-ETO*-expressing progenitors is shared with an embryonic population of cells containing the first few definitive human HSCs (Ivanovs et al., 2014).

Balanced levels of RUNX1-ETO expression (1) do not block blood formation; (2) confer survival, but not proliferation, to a subset of progenitors; (3) do not cause apoptosis (data not shown); and (4) maintain cells in a quiescent stage. Moreover, the latent colony-forming activity of progenitors that had been previously rendered quiescent by RUNX1-ETO induction re-emerges upon removal of Dox from the methylcellulose CFU assay. These cells even exhibit a higher yield of colonies when compared with their uninduced counterparts, suggesting that cells with high replating activity accumulate in induced cultures and that the blocked differentiation state is reversible, even after extended *RUNX1-ETO* expression. This result is consistent with prior replating assays of *RUNX1-ETO*-expressing mouse bone marrow cells, which

showed an increase of self-renewal, but not proliferation (Rhoades et al., 2000). Our observations are also consistent with the idea of the existence of a reservoir of cells harboring the t(8;21) translocation in a quiescent pre-leukemic state, as reported by the presence of a small population of HSCs harboring the translocation in t(8;21) patients in remission (Shima et al., 2014). In contrast, studies using retroviral transduction of human CD34+ cord blood hematopoietic cells to constitutively express *RUNX1-ETO* reported enhanced *in vitro* proliferation of these cells, while maintaining self-renewal and differentiation capacity (Mulloy et al., 2002, 2003). These discrepancies might be explained by differences in the expression levels of *RUNX1-ETO* used and/or by selection of specific clones of cells that were outgrowing in culture (Mulloy et al., 2002, 2003), indicating that the growth arrest can be surpassed. A dual role of RUNX1-ETO in blocking differentiation and arresting cell growth has been previously described (Burel et al., 2001), although those authors observed that RUNX1-ETO-dependent growth arrest resulted in apoptosis. Those studies were conducted in a cell line (U937) representing a different type of AML, and probably carrying additional oncogenic mutations, suggesting that RUNX1-ETO cannot reprogram one leukemic cell type into another. This observation is consistent with previous work from our lab that showed that each leukemogenic mutation directs hematopoietic precursors through a different developmental path (Assi et al., 2019).

RUNX1-ETO Induction Causes Extensive Global Chromatin Reorganization and Shuts Down the RUNX1-Directed Gene Expression Program

We found that the pattern of up- and downregulated RUNX1-ETO target genes is tightly correlated to changes observed in t(8;21) patient cells. Differential gene expression analyses upon induction of RUNX1-ETO showed downregulation of myelopoiesis, cell-cycle, and DNA repair genes. This finding is consistent with the expression of surface markers characteristic of immature cells in RUNX1-ETO-induced progenitors and the lack of proliferation of such cells. Interestingly, RUNX1-ETO induction caused an upregulation of genes from multiple signaling pathways, such as the MAPK pathway. General signaling pathway activation could occur as a survival response from the cells trying to compensate for the RUNX1-ETO-mediated block on the cell cycle. This hypothesis is supported by data from established t(8;21) AML cells that show that high *CDK6* and *CCND1/2* expression levels depend on the AP-1 transcription factor family, which mediates MAPK signaling and whose expression, in turn, depends on the presence of RUNX1-ETO (Martinez-Soria et al., 2019). Moreover, AP-1 is a critical factor for the establishment of t(8;21) AML in xenotransplantation experiments (Assi et al., 2019). To develop overt disease, RUNX1-ETO cooperates with mutated growth factor receptors, such as KIT or FLT3 (Schessl et al., 2005; Wichmann et al., 2015), indicating that such mutations may act upon an already partially activated signaling landscape.

Changes in gene expression after induction of RUNX1-ETO were mediated by rapid reorganization of the chromatin landscape, leading to loss of accessibility of thousands of sites enriched in RUNX motifs and bound by RUNX1, although *RUNX1* expression per se was not affected. Concomitantly, H3K27ac was reduced, demonstrating that binding of RUNX1-ETO directly interferes with that of RUNX1. It has been shown that t(8;21) cells depend on the presence of a normal *RUNX1* allele, which is required to balance the detrimental effects of *RUNX1-ETO* expression by upregulating critical mitotic checkpoint genes (Ben-Ami et al., 2013; Loke et al., 2017). It is therefore possible that restoring the growth of RUNX1-ETO-induced cells requires the re-establishment of at least part of the RUNX1-mediated gene expression program. This is likely to include the expression of *SPI1* (PU.1) (Huang et al., 2008) or *CEBPA*, as well as genes that override the RUNX1-ETO-mediated cell-cycle block.

RUNX1-ETO Expression Blocks Myeloid Differentiation by Rapidly Downregulating *SPI1* and *CEBPA* Expression in Early Myeloid Cells

Reprogramming of one cell fate into another requires a complete reorganization of the epigenome and is facilitated by reprogramming cells within a similar developmental pathway (Graf and Enver, 2009), but it can also be enforced upon cells from different pathways by overexpression of complementary transcription factors (Takahashi and Yamanaka, 2006). One of the most important and unanswered questions in AML research is the understanding of the nature of the target cells that are susceptible to an oncogenic hit. This notion is exemplified by our finding that RUNX1C− and RUNX1C+ cells significantly differ in their response to the expression of the fusion protein. Our scRNA-seq data clearly demonstrate that only early multipotent myeloid precursors respond to RUNX1-ETO induction with a block in differentiation and growth arrest. We do not know where these cells exist in human embryos, only that they suffer strong downregulation of the myeloid master regulator PU.1, without which myelopoiesis is strongly perturbed (McKercher et al., 1996; Scott et al., 1994), providing a molecular explanation for the RUNX1-ETO-mediated differentiation block. Expression of *RUNX1-ETO* also leads to an upregulation of *SOX4* in the 5-Dox-enriched cell cluster, which is consistent with the downregulation of *CEBPA*. *SOX4* expression is required for self-renewal of HSCs, as well as leukemic stem cells (LSCs), and its expression has been shown to be upregulated in HSCs from *CEBPA* null mice and in patients with abnormal C/EBPα function (Zhang et al., 2013) and thus myelopoiesis.

In summary, we have shown that we can use the differentiation of human ESCs into definitive hematopoietic progenitor cells to gain insight into the earliest events of the reprogramming of the myeloid gene regulatory network by RUNX1-ETO and its interplay with RUNX1 in a human embryonic setting. Our future experiments are aimed at further understanding the nature of the growth stimulus required to overcome RUNX1-ETO-mediated growth arrest.

STAR★METHODS

Detailed methods are provided in the online version of this paper and include the following:

- KEY RESOURCES TABLE
- RESOURCE AVAILABILITY
  - Lead contact
  - Materials availability
  - Data and code availability

- EXPERIMENTAL MODEL AND SUBJECT DETAILS
  - Generation and validation of targeted inducible RUNX1-ETO human ESC lines
  - Maintenance of human ESC cultures
  - *In vitro* hematopoietic differentiation of human ESC
- METHOD DETAILS
  - Flow cytometric analysis
  - Cell sorting
  - Intracellular immunostaining
  - Imaging
  - Colony-forming assays
  - Replating assays
  - Cell cycle analysis
  - Western Blotting
  - Gene expression analysis
  - RNA-Seq library preparation
  - Assay for transposase-accessible chromatin with high-throughput sequencing (ATAC-seq)
  - Chromatin Immunoprecipitation with high-throughput sequencing (ChIP-Seq)
  - Single Cell RNA-Seq (scRNA-Seq)
- QUANTIFICATION AND STATISTICAL ANALYSIS
  - Statistical analysis
  - Bulk RNA-Seq data analysis
  - ATAC-Seq data analysis
  - ChIP-Seq data analysis
  - Construction of average profiles
  - Single cell RNA-Seq data analysis

SUPPLEMENTAL INFORMATION

Supplemental Information can be found online at https://doi.org/10.1016/j.celrep.2020.107691.

ACKNOWLEDGMENTS

This work was funded by a U21 Melbourne studentship and a Wellcome Trust Institutional Strategic Support Fund (ISSF) grant to the University of Birmingham held by M.N., as well as a grant from the Birmingham Cancer Research UK (CRUK) Centre to C.B. We thank the Birmingham Next Generation Sequencing Facility (Genomics Birmingham) for expert service. M.N. was awarded The Henry and Rachael Ackman Travelling Scholarship by the University of Melbourne Faculty of Medicine, Dentistry and Health Sciences. Work in the laboratories of A.G.E. and E.G.S. was funded by the National Health and Medical Research Council of Australia (GNT1068866, GNT1129861, and GNT1138717), the Australian Research Council Special Research Initiative in Stem Cells (Stem Cells Australia), the Children's Cancer Foundation, and the Stafford Fox Medical Research Foundation. E.G.S. (GNT1079004) and A.G.E. (GNT1117596) are research fellows of National Health and Medical Research Council (Australia). Additional infrastructure funding to the Murdoch Children's Research Institute was provided by the Australian Government National Health and Medical Research Council Independent Research Institute Infrastructure Support Scheme and the Victorian government's Operational Infrastructure Support Program.

AUTHOR CONTRIBUTIONS

M.N. performed experiments and wrote the paper, P.K. analyzed the data, E.S.N. and E.G.S. helped with experiments, and A.G.E. and C.B. conceived the study and wrote the paper.

DECLARATION OF INTERESTS

The authors declare no competing interests.

Received: September 24, 2019
Revised: March 12, 2020
Accepted: May 5, 2020
Published: May 26, 2020

REFERENCES

Assi, S.A., Imperato, M.R., Coleman, D.J.L., Pickin, A., Potluri, S., Ptasinska, A., Chin, P.S., Blair, H., Cauchy, P., James, S.R., et al. (2019). Subtype-specific regulatory network rewiring in acute myeloid leukemia. Nat. Genet. *51*, 151–162.

Ben-Ami, O., Friedman, D., Leshkowitz, D., Goldenberg, D., Orlovsky, K., Pencovich, N., Lotem, J., Tanay, A., and Groner, Y. (2013). Addiction of t(8;21) and inv(16) acute myeloid leukemia to native RUNX1. Cell Rep. *4*, 1131–1143.

Bertrand, J.Y., Chi, N.C., Santoso, B., Teng, S., Stainier, D.Y.R., and Traver, D. (2010). Haematopoietic stem cells derive directly from aortic endothelium during development. Nature *464*, 108–111.

Bindea, G., Mlecnik, B., Hackl, H., Charoentong, P., Tosolini, M., Kirilovsky, A., Fridman, W.-H., Pagès, F., Trajanoski, Z., and Galon, J. (2009). ClueGO: a Cytoscape plug-in to decipher functionally grouped gene ontology and pathway annotation networks. Bioinformatics *25*, 1091–1093.

Boisset, J.-C., van Cappellen, W., Andrieu-Soler, C., Galjart, N., Dzierzak, E., and Robin, C. (2010). *In vivo* imaging of haematopoietic cells emerging from the mouse aortic endothelium. Nature *464*, 116–120.

Bolger, A.M., Lohse, M., and Usadel, B. (2014). Trimmomatic: a flexible trimmer for Illumina sequence data. Bioinformatics *30*, 2114–2120.

Bomken, S., Buechler, L., Rehe, K., Ponthan, F., Elder, A., Blair, H., Bacon, C.M., Vormoor, J., and Heidenreich, O. (2013). Lentiviral marking of patient-derived acute lymphoblastic leukaemic cells allows *in vivo* tracking of disease progression. Leukemia *27*, 718–721.

Bonifer, C., and Cockerill, P.N. (2017). Chromatin priming of genes in development: Concepts, mechanisms and consequences. Exp. Hematol. *49*, 1–8.

Buenrostro, J.D., Wu, B., Chang, H.Y., and Greenleaf, W.J. (2015). ATAC-seq: A method for assaying chromatin accessibility genome-wide. Curr. Protoc. Mol. Biol. *109*, 21.29.1–21.29.9.

Burel, S.A., Harakawa, N., Zhou, L., Pabst, T., Tenen, D.G., and Zhang, D.E (2001). Dichotomy of AML1-ETO functions: growth arrest versus block of differentiation. Mol. Cell. Biol. *21*, 5577–5590.

Butler, A., Hoffman, P., Smibert, P., Papalexi, E., and Satija, R. (2018). Integrating single-cell transcriptomic data across different conditions, technologies, and species. Nat. Biotechnol. *36*, 411–420.

Cabezas-Wallscheid, N., Eichwald, V., de Graaf, J., Löwer, M., Lehr, H.-A., Kreft, A., Eshkind, L., Hildebrandt, A., Abassi, Y., Heck, R., et al. (2013). Instruction of haematopoietic lineage choices, evolution of transcriptional landscapes and cancer stem cell hierarchies derived from an AML1-ETO mouse model. EMBO Mol. Med. *5*, 1804–1820.

Clarke, R.L., Yzaguirre, A.D., Yashiro-Ohtani, Y., Bondue, A., Blanpain, C., Pear, W.S., Speck, N.A., and Keller, G. (2013). The expression of Sox17 identifies and regulates haemogenic endothelium. Nat. Cell Biol. *15*, 502–510.

Corces, M.R., Buenrostro, J.D., Wu, B., Greenside, P.G., Chan, S.M., Koenig, J.L., Snyder, M.P., Pritchard, J.K., Kundaje, A., Greenleaf, W.J., et al. (2016). Lineage-specific and single-cell chromatin accessibility charts human hematopoiesis and leukemia evolution. Nat. Genet. *48*, 1193–1203.

de Bruijn, M.F.T.R., Ma, X., Robin, C., Ottersbach, K., Sanchez, M.J., and Dzierzak, E. (2002). Hematopoietic stem cells localize to the endothelial cell layer in the midgestation mouse aorta. Immunity *16*, 673–683.

Drissen, R., Thongjuea, S., Theilgaard-Mönch, K., and Nerlov, C. (2019). Identification of two distinct pathways of human myelopoiesis. Sci. Immunol. *4*, eaau7148.

Graf, T., and Enver, T. (2009). Forcing cells to change lineages. Nature *462*, 587–594.

Heinz, S., Benner, C., Spann, N., Bertolino, E., Lin, Y.C., Laslo, P., Cheng, J.X., Murre, C., Singh, H., and Glass, C.K. (2010). Simple combinations of lineage-determining transcription factors prime *cis*-regulatory elements required for macrophage and B cell identities. Mol. Cell *38*, 576–589.

Higuchi, M., O'Brien, D., Kumaravelu, P., Lenny, N., Yeoh, E.-J., and Downing, J.R. (2002). Expression of a conditional AML1-ETO oncogene bypasses embryonic lethality and establishes a murine model of human t(8;21) acute myeloid leukemia. Cancer Cell *1*, 63–74.

Huang, G., Zhang, P., Hirai, H., Elf, S., Yan, X., Chen, Z., Koschmieder, S., Okuno, Y., Dayaram, T., Growney, J.D., et al. (2008). PU.1 is a major downstream target of AML1 (RUNX1) in adult mouse hematopoiesis. Nat. Genet. *40*, 51–60.

Ivanovs, A., Rybtsov, S., Anderson, R.A., Turner, M.L., and Medvinsky, A. (2014). Identification of the niche and phenotype of the first human hematopoietic stem cells. Stem Cell Reports *2*, 449–456.

Jaffredo, T., Gautier, R., Eichmann, A., and Dieterlen-Lièvre, F. (1998). Intraaortic hemopoietic cells are derived from endothelial cells during ontogeny. Development *125*, 4575–4583.

Karolchik, D., Hinrichs, A.S., Furey, T.S., Roskin, K.M., Sugnet, C.W., Haussler, D., and Kent, W.J. (2004). The UCSC Table Browser data retrieval tool. Nucleic Acids Res. *32*, D493–D496.

Kim, D., Langmead, B., and Salzberg, S.L. (2015). HISAT: a fast spliced aligner with low memory requirements. Nat. Methods *12*, 357–360.

Kissa, K., and Herbomel, P. (2010). Blood stem cells emerge from aortic endothelium by a novel type of cell transition. Nature *464*, 112–115.

Lam, E.Y.N., Hall, C.J., Crosier, P.S., Crosier, K.E., and Flores, M.V. (2010). Live imaging of Runx1 expression in the dorsal aorta tracks the emergence of blood progenitors from endothelial cells. Blood *116*, 909–914.

Langfelder, P., Zhang, B., and Horvath, S. (2008). Defining clusters from a hierarchical cluster tree: the Dynamic Tree Cut package for R. Bioinformatics *24*, 719–720.

Langmead, B., and Salzberg, S.L. (2012). Fast gapped-read alignment with Bowtie 2. Nat. Methods *9*, 357–359.

Leddin, M., Perrod, C., Hoogenkamp, M., Ghani, S., Assi, S., Heinz, S., Wilson, N.K., Follows, G., Schönheit, J., Vockentanz, L., et al. (2011). Two distinct auto-regulatory loops operate at the PU.1 locus in B cells and myeloid cells. Blood *117*, 2827–2838.

Loke, J., Assi, S.A., Imperato, M.R., Ptasinska, A., Cauchy, P., Grabovska, Y., Soria, N.M., Raghavan, M., Delwel, H.R., Cockerill, P.N., et al. (2017). RUNX1-ETO and RUNX1-EVI1 Differentially Reprogram the Chromatin Landscape in t(8;21) and t(3;21) AML. Cell Rep. *19*, 1654–1668.

Lutterbach, B., Westendorf, J.J., Linggi, B., Patten, A., Moniwa, M., Davie, J.R., Huynh, K.D., Bardwell, V.J., Lavinsky, R.M., Rosenfeld, M.G., et al. (1998). ETO, a target of t(8;21) in acute leukemia, interacts with the N-CoR and mSin3 corepressors. Mol. Cell. Biol. *18*, 7176–7184.

Mandoli, A., Singh, A.A., Prange, K.H.M., Tijchon, E., Oerlemans, M., Dirks, R., Ter Huurne, M., Wierenga, A.T.J., Janssen-Megens, E.M., Berentsen, K., et al. (2016). The Hematopoietic Transcription Factors RUNX1 and ERG Prevent AML1-ETO Oncogene Overexpression and Onset of the Apoptosis Program in t(8;21) AMLs. Cell Rep. *17*, 2087–2100.

Martens, J.H., Mandoli, A., Simmer, F., Wierenga, B.J., Saeed, S., Singh, A.A., Altucci, L., Vellenga, E., and Stunnenberg, H.G. (2012). ERG and FLI1 binding sites demarcate targets for aberrant epigenetic regulation by AML1-ETO in acute myeloid leukemia. Blood *120*, 4038–4048.

Martinez-Soria, N., McKenzie, L., Draper, J., Ptasinska, A., Issa, H., Potluri, S., Blair, H.J., Pickin, A., Isa, A., Chin, P.S., et al. (2019). The Oncogenic Transcription Factor RUNX1/ETO Corrupts Cell Cycle Regulation to Drive Leukemic Transformation. Cancer Cell *35*, 705.

McKercher, S.R., Torbett, B.E., Anderson, K.L., Henkel, G.W., Vestal, D.J., Baribault, H., Klemsz, M., Feeney, A.J., Wu, G.E., Paige, C.J., and Maki, R.A. (1996). Targeted disruption of the PU.1 gene results in multiple hematopoietic abnormalities. EMBO J. *15*, 5647–5658.

Miyamoto, T., Weissman, I.L., and Akashi, K. (2000). AML1/ETO-expressing nonleukemic stem cells in acute myelogenous leukemia with 8;21 chromosomal translocation. Proc. Natl. Acad. Sci. USA *97*, 7521–7526.

Miyoshi, H., Shimizu, K., Kozu, T., Maseki, N., Kaneko, Y., and Ohki, M. (1991). t(8;21) breakpoints on chromosome 21 in acute myeloid leukemia are clustered within a limited region of a single gene, AML1. Proc. Natl. Acad. Sci. USA *88*, 10431–10434.

Mulloy, J.C., Cammenga, J., MacKenzie, K.L., Berguido, F.J., Moore, M.A., and Nimer, S.D. (2002). The AML1-ETO fusion protein promotes the expansion of human hematopoietic stem cells. Blood *99*, 15–23.

Mulloy, J.C., Cammenga, J., Berguido, F.J., Wu, K., Zhou, P., Comenzo, R.L., Jhanwar, S., Moore, M.A., and Nimer, S.D. (2003). Maintaining the self-renewal and differentiation potential of human CD34+ hematopoietic cells using a single genetic element. Blood *102*, 4369–4376.

Ng, E.S., Davis, R., Stanley, E.G., and Elefanty, A.G. (2008). A protocol describing the use of a recombinant protein-based, animal product-free medium (APEL) for human embryonic stem cell differentiation as spin embryoid bodies. Nat. Protoc. *3*, 768–776.

Ng, E.S., Azzola, L., Bruveris, F.F., Calvanese, V., Phipson, B., Vlahos, K., Hirst, C., Jokubaitis, V.J., Yu, Q.C., Maksimovic, J., et al. (2016). Differentiation of human embryonic stem cells to HOXA^{+} hemogenic vasculature that resembles the aorta-gonad-mesonephros. Nat. Biotechnol. *34*, 1168–1179.

North, T.E., de Bruijn, M.F.T.R., Stacy, T., Talebian, L., Lind, E., Robin, C., Binder, M., Dzierzak, E., and Speck, N.A. (2002). Runx1 expression marks long-term repopulating hematopoietic stem cells in the midgestation mouse embryo. Immunity *16*, 661–672.

O'Leary, N.A., Wright, M.W., Brister, J.R., Ciufo, S., Haddad, D., McVeigh, R., Rajput, B., Robbertse, B., Smith-White, B., Ako-Adjei, D., et al. (2016). Reference sequence (RefSeq) database at NCBI: current status, taxonomic expansion, and functional annotation. Nucleic Acids Res. *44* (*D1*), D733–D745.

Obier, N., Cauchy, P., Assi, S.A., Gilmour, J., Lie-A-Ling, M., Lichtinger, M., Hoogenkamp, M., Noailles, L., Cockerill, P.N., Lacaud, G., et al. (2016). Cooperative binding of AP-1 and TEAD4 modulates the balance between vascular smooth muscle and hemogenic cell fate. Development *143*, 4324–4340.

Pertea, M., Pertea, G.M., Antonescu, C.M., Chang, T.-C., Mendell, J.T., and Salzberg, S.L. (2015). StringTie enables improved reconstruction of a transcriptome from RNA-seq reads. Nat. Biotechnol. *33*, 290–295.

Ptasinska, A., Assi, S.A., Mannari, D., James, S.R., Williamson, D., Dunne, J., Hoogenkamp, M., Wu, M., Care, M., McNeill, H., et al. (2012). Depletion of RUNX1/ETO in t(8;21) AML cells leads to genome-wide changes in chromatin structure and transcription factor binding. Leukemia *26*, 1829–1841.

Ptasinska, A., Assi, S.A., Martinez-Soria, N., Imperato, M.R., Piper, J., Cauchy, P., Pickin, A., James, S.R., Hoogenkamp, M., Williamson, D., et al. (2014). Identification of a dynamic core transcriptional network in t(8;21) AML that regulates differentiation block and self-renewal. Cell Rep. *8*, 1974–1988.

Ptasinska, A., Pickin, A., Assi, S.A., Chin, P.S., Ames, L., Avellino, R., Gröschel, S., Delwel, R., Cockerill, P.N., Osborne, C.S., and Bonifer, C. (2019). RUNX1-ETO Depletion in t(8;21) AML Leads to C/EBPα- and AP-1-Mediated Alterations in Enhancer-Promoter Interaction. Cell Rep. *28*, 3022–3031.e7.

Qian, K., Huang, C.T.-L., Chen, H., Blackbourn, L.W., 4th, Chen, Y., Cao, J., Yao, L., Sauvey, C., Du, Z., and Zhang, S.C. (2014). A simple and efficient system for regulating gene expression in human pluripotent stem cells and derivatives. Stem Cells *32*, 1230–1238.

Qiu, X., Mao, Q., Tang, Y., Wang, L., Chawla, R., Pliner, H.A., and Trapnell, C. (2017). Reversed graph embedding resolves complex single-cell trajectories. Nat. Methods *14*, 979–982.

Ramírez, F., Ryan, D.P., Grüning, B., Bhardwaj, V., Kilpert, F., Richter, A.S., Heyne, S., Dündar, F., and Manke, T. (2016). deepTools2: a next generation web server for deep-sequencing data analysis. Nucleic Acids Res. *44* (*W1*), W160–W165.

Regha, K., Assi, S.A., Tsoulaki, O., Gilmour, J., Lacaud, G., and Bonifer, C. (2015). Developmental-stage-dependent transcriptional response to leukaemic oncogene expression. Nat. Commun. *6*, 7203.

Rhoades, K.L., Hetherington, C.J., Harakawa, N., Yergeau, D.A., Zhou, L., Liu, L.Q., Little, M.T., Tenen, D.G., and Zhang, D.E. (2000). Analysis of the role of AML1-ETO in leukemogenesis, using an inducible transgenic mouse model. Blood *96*, 2108–2115.

Ritchie, M.E., Phipson, B., Wu, D., Hu, Y., Law, C.W., Shi, W., and Smyth, G.K. (2015). limma powers differential expression analyses for RNA-sequencing and microarray studies. Nucleic Acids Res. *43*, e47.

Saldanha, A.J. (2004). Java Treeview—extensible visualization of microarray data. Bioinformatics *20*, 3246–3248.

Schessl, C., Rawat, V.P.S., Cusan, M., Deshpande, A., Kohl, T.M., Rosten, P.M., Spiekermann, K., Humphries, R.K., Schnittger, S., Kern, W., et al. (2005). The AML1-ETO fusion gene and the FLT3 length mutation collaborate in inducing acute leukemia in mice. J. Clin. Invest. *115*, 2159–2168.

Scott, E., Simon, M., Anastasi, J., and Singh, H. (1994). Requirement of transcription factor PU.1 in the development of multiple hematopoietic lineages. Science *265*, 1573–1577.

Shannon, P., Markiel, A., Ozier, O., Baliga, N.S., Wang, J.T., Ramage, D., Amin, N., Schwikowski, B., and Ideker, T. (2003). Cytoscape: a software environment for integrated models of biomolecular interaction networks. Genome Res. *13*, 2498–2504.

Shima, T., Miyamoto, T., Kikushige, Y., Yuda, J., Tochigi, T., Yoshimoto, G., Kato, K., Takenaka, K., Iwasaki, H., Mizuno, S., et al. (2014). The ordered acquisition of Class II and Class I mutations directs formation of human t(8;21) acute myelogenous leukemia stem cell. Exp. Hematol. *42*, 955–965.e1–e5.

Sroczynska, P., Lancrin, C., Kouskoff, V., and Lacaud, G. (2009). The differential activities of Runx1 promoters define milestones during embryonic hematopoiesis. Blood *114*, 5279–5289.

Subramanian, A., Tamayo, P., Mootha, V.K., Mukherjee, S., Ebert, B.L., Gillette, M.A., Paulovich, A., Pomeroy, S.L., Golub, T.R., Lander, E.S., and Mesirov, J.P. (2005). Gene set enrichment analysis: a knowledge-based approach for interpreting genome-wide expression profiles. Proc. Natl. Acad. Sci. USA *102*, 15545–15550.

Sun, X.-J., Wang, Z., Wang, L., Jiang, Y., Kost, N., Soong, T.D., Chen, W.-Y., Tang, Z., Nakadai, T., Elemento, O., et al. (2013). A stable transcription factor complex nucleated by oligomeric AML1-ETO controls leukaemogenesis. Nature *500*, 93–97.

Takahashi, K., and Yamanaka, S. (2006). Induction of pluripotent stem cells from mouse embryonic and adult fibroblast cultures by defined factors. Cell *126*, 663–676.

Tijchon, E., Yi, G., Mandoli, A., Smits, J.G.A., Ferrari, F., Heuts, B.M.H., Wijnen, F., Kim, B., Janssen-Megens, E.M., Schuringa, J.J., and Martens, J.H.A. (2019). The acute myeloid leukemia associated AML1-ETO fusion protein alters the transcriptome and cellular progression in a single-oncogene expressing *in vitro* induced pluripotent stem cell based granulocyte differentiation model. PLoS ONE *14*, e0226435.

Trapnell, C., Cacchiarelli, D., Grimsby, J., Pokharel, P., Li, S., Morse, M., Lennon, N.J., Livak, K.J., Mikkelsen, T.S., and Rinn, J.L. (2014). The dynamics and regulators of cell fate decisions are revealed by pseudotemporal ordering of single cells. Nat. Biotechnol. *32*, 381–386.

Wichmann, C., Quagliano-Lo Coco, I., Yildiz, Ö., Chen-Wichmann, L., Weber, H., Syzonenko, T., Döring, C., Brendel, C., Ponnusamy, K., Kinner, A., et al. (2015). Activating c-KIT mutations confer oncogenic cooperativity and rescue RUNX1/ETO-induced DNA damage and apoptosis in human primary CD34+ hematopoietic progenitors. Leukemia *29*, 279–289.

Wiemels, J.L., Xiao, Z., Buffler, P.A., Maia, A.T., Ma, X., Dicks, B.M., Smith, M.T., Zhang, L., Feusner, J., Wiencke, J., et al. (2002). *In utero* origin of t(8;21) AML1-ETO translocations in childhood acute myeloid leukemia. Blood *99*, 3801–3805.

Yergeau, D.A., Hetherington, C.J., Wang, Q., Zhang, P., Sharpe, A.H., Binder, M., Marín-Padilla, M., Tenen, D.G., Speck, N.A., and Zhang, D.E. (1997). Embryonic lethality and impairment of haematopoiesis in mice heterozygous for an AML1-ETO fusion gene. Nat. Genet. *15*, 303–306.

Yuan, Y., Zhou, L., Miyamoto, T., Iwasaki, H., Harakawa, N., Hetherington, C.J., Burel, S.A., Lagasse, E., Weissman, I.L., Akashi, K., and Zhang, D.E. (2001). AML1-ETO expression is directly involved in the development of acute myeloid leukemia in the presence of additional mutations. Proc. Natl. Acad. Sci. USA *98*, 10398–10403.

Zhang, Y., Liu, T., Meyer, C.A., Eeckhoute, J., Johnson, D.S., Bernstein, B.E., Nusbaum, C., Myers, R.M., Brown, M., Li, W., and Liu, X.S. (2008). Model-based analysis of ChIP-Seq (MACS). Genome Biol. *9*, R137.

Zhang, H., Alberich-Jorda, M., Amabile, G., Yang, H., Staber, P.B., Di Ruscio, A., Welner, R.S., Ebralidze, A., Zhang, J., Levantini, E., et al. (2013). Sox4 is a key oncogenic target in C/EBPα mutant acute myeloid leukemia. Cancer Cell *24*, 575–588.

STAR★METHODS

KEY RESOURCES TABLE

REAGENT or RESOURCE	SOURCE	IDENTIFIER
Antibodies		
Anti-HA tag (Abcam)	Sigma	Cat# H6908; RRID: AB_260070
Anti-Histone H3 (Rabbit polyclonal)	Abcam	Cat# ab1791; RRID: AB_302613
Anti-Histone H3K27ac	Abcam	Cat# ab4729; RRID: AB_2118291
Anti-Histone H3K4me3	Merk	Cat# 07-473; RRID: AB_1977252
Anti-human AML1 (Rabbit Polyclonal)	Cell Signaling Technology	Cat# 4334; RRID: AB_2184099
Anti-LDB1	Abcam	Cat# ab96799; RRID: AB_10679400
Anti-LMO2	R&D Systems	Cat# AF2726; RRID: AB_2249968
Anti-RUNX1	Abcam	Cat# ab23980; RRID: AB_2184205
APC anti-human CD38	BD PharMingen	Cat# 555462; RRID: AB_398599
APC anti-human CD90	BD PharMingen	Cat# 559869; RRID: AB_398677
BV-421 anti-human CD326 (EpCam)	BioLegend	Cat# 324220; RRID: AB_2563847
BV-421 anti-human CD34	BioLegend	Cat# 343609; RRID: AB_11147951
BV-421 anti-human CD45	BioLegend	Cat# 304032; RRID: AB_2561357
BV-421 anti-human CD90	BioLegend	Cat# 328121; RRID: AB_10933261
DyLight® 650-conjugated anti-HA tag [16B12]	Abcam	Cat# ab117515; RRID: AB_10999718
FITC-conjugated BrdU and IgG	BD PharMingen	Cat# 556028; RRID: AB_396304
PE anti-human CD9	BD PharMingen	Cat# 555372; RRID: AB_395774
PeCy7 anti-human CD16	BioLegend	Cat# 302015; RRID: AB_314215
PeCy7 anti-human CD34	BioLegend	Cat# 343516; RRID: AB_1877251
Bacterial and Virus Strains		
One Shot™ TOP10 Chemically Competent *E. Coli*	Thermo Fisher Scientific	Cat# C404010
Chemicals, Peptides, and Recombinant Proteins		
2-Mercaptoethanol	Thermo Fisher Scientific	Cat# 21985-023
a- Monothioglycerol (MTG)	Merck	Cat# M6145
Accutase solution	Merk	Cat# A6964
Activin A (ACT)	R&D Systems	Cat# 338-AC
Albumin from rice endosperm	ScienCell Research Labs	Cat# OsSA
AMPure magnetic beads	Beckman Coulter	Cat# A63881
Ascorbic acid 2-phosphate (AA2P)	Merck	Cat# A8960
Bone morphogenetic protein 4 (BMP4)	R&D Systems	Cat# 314-BP
Bovostar acid-stripped Bovine Serum Albumin (BSA)	Bovogen	Cat# BSAS 0.1
BrdU	Sigma	Cat# B5002
CHIR99021	Tocris Biosciences	Cat# 4423
Collagenase Type 4	Worthington	Cat# CLS-4
Digitonin	Promega	Cat# G9441
Dimethyl sulfoxide Hybri-MaxTM (DMSO)	Merk	Cat# D2650
DMEM Nutrient Mixture F- 12 1x + L-glutamine Na bicarbonate (DMEM/F12)	Thermo Fisher Scientific	Cat# 11320-033
Erythropoietin (EPO)	PeproTech	Cat# 100-64
Fibroblast Growth Factor (FGF2)	PeproTech	Cat# 100-18B
Fixation/Permeabilization solution	BD PharMingen	Cat# 554722

(*Continued on next page*)

Continued

REAGENT or RESOURCE	SOURCE	IDENTIFIER
FMS-like tyrosine kinase 3 receptor (FLT3) ligand	PeproTech	Cat# 300-19
GlutaMAXI100x	Thermo Fisher Scientific	Cat# 35050-061
Ham's F12	Thermo Fisher Scientific	Cat# 11765-062
Human low-density lipoproteins (hLDL)	Stem Cell Technologies	Cat# 02698
Insulin-like growth factor 2 (IGF2)	PeproTech	Cat# 100-12
Insulin-Transferrin-Selenium-E (ITS-E)	In Vitria	Cat# 777ITS092
Interleukin 3 (IL-3)	PeproTech	Cat# 200-03
Interleukin 6 (IL-6)	PeproTech	Cat# 200-06
Iscove's Modified Dulbecco's Media (IMDM) with no phenol red	Themo Fisher Scientific	Cat# 21056-023
KnockOut Serum Replacer (KO-SR)	Thermo Fisher Scientific	Cat# 10828028
L-Ascorbic acid	Merck	Cat# A4403
Linoleic acid	Merck	Cat# L2376
Linolenic acid	Merck	Cat# L1376
Matrigel Growth Factor Reduced phenol red-free	*In Vitro* Technologies	Cat# FAL356231
Methanol-free Formaldehyde	Thermo Fisher Scientific	Cat# 28906
MethoCult™ H4100	Stem Cell Technologies	Cat# 01400
NEBNext High-Fidelity 2x PCR Master Mix	New England Biolabs	Cat# M0541
NextSeq® 500/550 High Output 150 cycle sequencing kit v2	Illumina	Cat# FC-404-2002
NextSeq® 500/550 High Output 75 cycle sequencing kit v2	Illumina	Cat# FC-404-2005
Non-essential amino acids (NEAA) 100X	Thermo Fisher Scientific	Cat# 11140-050
Penicillin/Streptomycin (Pen/Strep)	Thermo Fisher Scientific	Cat# 15140122
Perm/Wash buffer	BD PharMingen	Cat# 554723
Polyvinyl alcohol (PVA)	Merck	Cat# P8136
Protein Free Hybridoma Medium II (PFHMII)	Thermo Fisher Scientific	Cat# 12040077
SB431542	Sapphire Bioscience	Cat# 13031
Soybean Oil (lecithin)	Merck	Cat# S7381
Stem cell factor (SCF)	PeproTech	Cat# 300-07
SuperScript II Reverse Transcriptase	Thermo Fisher Scientific	Cat# 18064022
SyntheChol	Merck	Cat# S5442
Thrombopoietin (TPO)	PeproTech	Cat# 300-18
TrypLE™ Select Enzyme	Thermo Fisher Scientific	Cat# 12563011
Vascular endothelial growth factor (VEGF)	PeproTech	Cat# 100-20
Vybrant DyeCycle Violet Stain	Thermo Fisher Scientific	Cat# V35003
Critical Commercial Assays		
Bioline Isolate II RNA Mini Kit	Bioline	Cat# BIO-52072
CD34 UltraPure human MicroBeads Kit	Miltenyi Biotec	Cat# 130-100-453
Chromium Single Cell 3′ Library and Gel Bead Kit v2	10X Genomics	Cat# PN-120237
High Sensitivity DNA Chip	Agilent Technologies	Cat# 5067-4626
KAPA Hyper Prep Kit	Roche	Cat# KR0961
KAPA Library Quantification Kit Illumina Sequencing Platforms	Roche	Cat# KR0405
MACS Starting Kit	Miltenyi Biotec	Cat# 130-091-632
MinElute Reaction Cleanup Kit	QIAGEN	Cat# 28204
Nextera DNA Library Prep Kit	Illumina	Cat# FC-121-1030

(*Continued on next page*)

Continued

REAGENT or RESOURCE	SOURCE	IDENTIFIER
Pierce BCA Protein Assay Kit	Thermo Fisher Scientific	Cat# 23225
SuperSignal PICO reagent mix	Thermo Fisher Scientific	Cat# 34579
Tetro cDNA synthesis kit	Bioline	Cat# BIO-65042
TruSeq® Stranded mRNA Library Prep	Illumina	Cat# 20020594
Universal Magnetic Co-IP Kit	Active Motif	Cat# 54002
Deposited Data		
Bulk RNA-seq data	This study	GEO: GSE137673
ChIP-seq data	This study	GEO: GSE137673
ATAC-seq data	This study	GEO: GSE137673
scRNA-seq data	This study	GEO: GSE137673
Experimental Models: Cell Lines		
SOX17$^{mCHERRY/w}$RUNX1C$^{GFP/w}$ hESC H9 line	Ng et al., 2016	N/A
Inducible RUNX1-ETO SOX17$^{mCHERRY/w}$RUNX1C$^{GFP/w}$ hESC H9 line	This paper	N/A
Oligonucleotides		
Oligonucleotide sequences, see Table S1	This paper	N/A
GAPDH (TaqMan assay)	Thermo Fisher Scientific	RRID: Hs99999905_m1
GATA1 (TaqMan assay)	Thermo Fisher Scientific	RRID: HS00231112_m1
GFI1 (TaqMan assay)	Thermo Fisher Scientific	RRID: Hs00382207_m1
GFI1B (TaqMan assay)	Thermo Fisher Scientific	RRID: Hs01062469_m1
SPI1 (PU.1) (TaqMan assay)	Thermo Fisher Scientific	RRID: HS00231368_m1
RUNX1 COMMON (TaqMan assay)	Thermo Fisher Scientific	RRID: HS00231079_m1
RUNX1C (TaqMan assay)	Thermo Fisher Scientific	RRID: Hs01021967_m1
RUNX1T1 (TaqMan assay)	Thermo Fisher Scientific	RRID: Hs00231702_m1
Recombinant DNA		
pSIEW-RUNX1-ETO	Bomken et al., 2013	N/A
pTREG-CAGGS-Tet3G-AAVS1	Qian et al., 2014	N/A
Software and Algorithms		
Trimmomatic v0.32	Bolger et al., 2014	http://www.usadellab.org/cms/?page=trimmomatic
Bowtie2 v2.2.6	Langmead and Salzberg., 2012	http://bowtie-bio.sourceforge.net/index.shtml
MACS2 v2.1.1	Zhang et al., 2008	https://github.com/taoliu/MACS
Picard v2.10.5	http://broadinstitute.github.io/picard	N/A
Homer v4.9.1	Heinz et al., 2010	http://homer.ucsd.edu/homer/
R v3.5.1	https://www.R-project.org/	N/A
Java TreeView v1.1	Saldanha, 2004	http://jtreeview.sourceforge.net/
deepTools v3.2.0	Ramírez et al., 2016	https://github.com/deeptools/deepTools
Hisat2 v2.1.0	Kim et al., 2015	https://daehwankimlab.github.io/hisat2/
Stringtie v1.3.3	Pertea et al., 2015	https://ccb.jhu.edu/software/stringtie/
Limma v3.26.9	Ritchie et al., 2015	https://bioconductor.org/packages/release/bioc/html/limma.html
Cytoscape v3.6.1	Shannon et al., 2003	https://cytoscape.org/
ClueGO v2.5.0	Bindea et al., 2009	http://apps.cytoscape.org/apps/cluego
dynamicTreeCut v1.63	Langfelder et al., 2008	https://cran.r-project.org/web/packages/dynamicTreeCut/index.html
GSEA v2.2.4	Subramanian et al., 2005	https://www.gsea-msigdb.org/gsea/index.jsp
CellRanger v2.1.1	https://www.10xgenomics.com/	https://support.10xgenomics.com/single-cell-gene-expression/software/downloads/latest

(*Continued on next page*)

CellPress
OPEN ACCESS

Continued

REAGENT or RESOURCE	SOURCE	IDENTIFIER
Seurat v2.3.4	Butler et al., 2018	https://satijalab.org/seurat/
Monocle v2.10.1	Qiu et al., 2017; Trapnell et al., 2014	http://cole-trapnell-lab.github.io/monocle-release/
Other		
Low attachment 96-well plates (Sterile)	Costar	Cat# COR3788
Ultra-low attachment 24-well plates	*In Vitro* AS	Cat# NUN144530
4-20% gradient polyacrylamide gel	Biorad	Cat# 456-8093
AML patient RNA-seq data	Assi et al., 2019	GEO: GSE108316
Hematopoietic progenitor RNA-seq data	Corces et al., 2016	GEO: GSE74912

RESOURCE AVAILABILITY

Lead contact

Further information and requests for resources and reagents should be directed to and will be fulfilled by the Lead Contact, Constanze Bonifer (c.bonifer@bham.ac.uk).

Materials availability

The targeting plasmid and the inducible RUNX1-ETO cell lines generated in this study are available from the Lead Contact.

Data and code availability

All high throughput data (bulk RNA-seq, ChIP-seq, ATAC-sec and scRNA-seq data) generated in this study are available at NCBI under the accession number GEO: GSE137673.

The published article includes AML patient RNA-seq data (Assi et al., 2019) with GEO: GSE108316 and hematopoietic progenitor RNA-seq data (Corces et al., 2016) with GEO: GSE74912, analyzed during this study.

EXPERIMENTAL MODEL AND SUBJECT DETAILS

Generation and validation of targeted inducible RUNX1-ETO human ESC lines

The dual reporter SOX17$^{mCHERRY/w}$RUNX1C$^{GFP/w}$ human ESC H9 line was previously generated by us (Ng et al., 2016), by targeting mCHERRY into exon 1 of one allele of SOX17 and GFP into exon 1 of one allele of RUNX1. RUNX1-ETO cDNA was amplified from the pSIEW-RUNX1-ETO vector with primers including a HA tag sequence following the Kozak sequence containing the start codon and restriction endonuclease sites SalI (5′) and MluI (3′) for subsequent cloning into the multiple cloning site of the pTREG-CAGGS-Tet3G-AAVS1 knockin plasmid. The primers used for cloning are listed in the Key Resources Table. The pSIEW-RUNX1-ETO and the pTREG-CAGGS-Tet3G-AAVS1 vectors were gifts from Olaf Heidenreich (Newcastle, UK) (Bomken et al., 2013) and Su-Chun Zhang (Wisconsin, US) (Qian et al., 2014), respectively. RUNX1-ETO-AAVS1 targeting vectors comprised a 804-bp 5′ homology arm, a tetracycline-inducible promoter (TRE3G-CMV) driving expression of HA-RUNX1-ETO cDNA sequence, Puromycin N-acetyltransferase resistance cassette with gene expression to be driven by endogenous promoter after genomic insertion, a CAG promoter driving expression of a modified Tet3G reverse tetracycline-controlled transactivator (rtTA) and a 837-bp 3′ homology arm. Vectors were electroporated with a pair of AAVS1 Transcription Activator-Like Effector Nucleases (TALENs) into SOX17$^{mCHERRY/w}$RUNX1C$^{GFP/w}$ human ESC H9 cells, which were then selected for Puromycin-resistant colony growth. Single cell sorted clones were screened for transgene insertion by PCR using primers designed to amplify the boundaries of the genomic insertion. Homozygous or heterozygous targeting of the AAVS1 locus was identified with a pair of primers designed to cover both locus sides, resulting in fragment amplification only in presence of a wild-type allele. The primers used for screening targeted clones are listed in the Key Resources Table. Genomic integrity in all clones was confirmed using the Illumina HumanCytoSNP-12 v2.1 array.

Maintenance of human ESC cultures

Culture and enzymatic passaging of human ESC lines was conducted as previously reported (Ng et al., 2008). Briefly, human ESCs were routinely co-cultured with mitotically inactivated primary mouse embryonic fibroblasts in a defined serum-free media in a humidified incubator at 37°C with 5% CO2 and low (5%) O2 conditions. The filter-sterilized media consisted of DMEM/F12 supplemented with 20% KO-SR, 1x NEAA, 200 mM GlutaMAXTMI, 55 mM 2-Mercaptoethanol and 10 ng/ml FGF2. All cell centrifugations were done at 300 x g for 3 min at 4°C. Passaging of human ESC cultures was performed using TrypLE^{TM} Select Enzyme. Cells were cryopreserved in 10% DMSO and CJ2 solution, consisting of 20x Choline Chloride (382 mg/ml in dH_2O), 0.01 mM $CaCl_2.2H_2O$, 2.68 mM KCl, 1.47 mM KH_2PO_4, 6.54 mM $K_2HPO_4.3H_2O$, 0.5 mM $MgCl_2.6H_2O$ and 5.5 mM D-glucose in dH_2O.

In vitro hematopoietic differentiation of human ESC

Hematopoietic differentiation of human ESCs was performed with a modified protocol of the spin Embryo Body (EB) method in serum-free STAPEL medium (Ng et al., 2008) supplemented with recombinant human protein components, as reported (Ng et al., 2016). STAPEL medium consisted of a mixture of 50% IMDM, 50% Ham's F12 and 0.05% PVA, supplemented with: 0.5% albumin (1:1 mix of albumin from rice endosperm and Bovostar acid-stripped BSA), 1x PFHMII, 0.03% MTG, 2.2 μg/ml SyntheChol, 100 ng/ml Linolenic and Linoleic acids, 0.005 mg/ml Soybean Oil, 2 mM GlutaMAXI, 50 μg/ml AA2P, 50 μg/ml L-Ascorbic acid and 1x ITS-E. To set up the differentiation (day 0), human ESCs were harvested from a confluent (95%–98%) T75 flask without feeders using Accutase solution and mixed into 50 mL of STAPEL media supplemented with cytokines (herein referred as STAGE1): 20 ng/ml BMP4, 25 ng/ml VEGF, 25 ng/ml SCF, 7.5 ng/ml ACT, 10 ng/ml FGF2 and 0.5 μM CHIR99021. All human cytokines used for hematopoietic differentiation were recombinant. Following cell resuspension in STAGE1 media, 80 μL were distributed into the each of the 60 inner wells of 10 low attachment 96-well plates, pre-filled with 70 μL sterile ddH$_2$O in the outer wells. Cells were aggregated into EBs (60 EBs/plate) at the bottom of the wells by centrifugation. Plates were incubated at 37°C with 5% CO_2 and high (air levels) O_2 conditions. Approximately 4–6 h before the 48-hour time point from set up (day 1.6-1.7), 20 μL of STAPEL supplemented with 3.5 μM SB431542 and 3 μM CHIR99021 were added to each well. After day 4, STAGE 1 media was removed and 100 μL of STAGE2 STAPEL were added to each well. STAGE2 cytokines consisted of 5 ng/ml BMP4, 50 ng/ml VEGF, 50 ng/ml SCF, 10 ng/ml IGF2 and 10 ng/ml FGF2. At day 8, 20–30 EBs/well were transferred onto 6-well adherent plates pre-coated with Matrigel solution (IMDM with 1x Pen/Strep and 1:200 Corning® Matrigel® Growth Factor Reduced phenol red-free). Adherent cultures after d8 were fed with STAGE3 STAPEL supplemented with 20 ng/ml BMP4, 100 ng/ml SCF, 100 ng/ml FLT3, 50 ng/ml VEGF, 50 ng/ml TPO, 25 ng/ml IL-3, 25 ng/ml IL-6, 20 ng/ml IGF2, 10 ng/ml FGF2 and 1x Pen/Strep. Plates were toped up with media every 2–3 days and half-media changes were performed when the media capacity of the plate was reached. After progenitor formation (~d14), cultures were supplemented with a '5-factor' cytokine mix including 100 ng/ml SCF, 100 ng/ml FLT3 ligand, 50 ng/ml TPO, 25 ng/ml IL3 and 25 ng/ml IL-6. During the 7-day Dox treatment, Dox was refreshed at the half-media changes (every 2–3 days). For analysis, EBs were harvested at different time points and dissociated into single-cell suspensions using TryPLE select for non-adherent EBs (d7) and Collagenase Type 4 for adherent EB cultures and passed through 23- and 25-gauge needles and a 40 μm filter (Ng et al., 2008).

METHOD DETAILS

Flow cytometric analysis

Flow cytometric analysis was performed using BD Fortessa analyzer using antibodies against surface antigens detailed in the Key Resources Table. For intracellular flow cytometric analysis, cell pellets were fixed with Fixation/Permeabilization solution on ice for 30 min. Cell suspensions were washed with 1x Perm/Wash buffer (BD PharMingen cat# 554723) and HA-RUNX1-ETO was detected using a primary conjugated antibody against the HA-tag (Anti-HA tag [16B12] DyLight® 650-conjugated, Abcam cat# ab117515).

Cell sorting

Fluorescence-activated cell sorting (FACS) was done in a FACS Aria cell sorter. Antibodies against CD9 and EpCam were used for sorting undifferentiated human ESCs and against CD34, CD45 and CD90 for sorting hematopoietic progenitors (Key Resources Table). Magnetic-activated cell sorting (MACS) of the CD34^{high} hematopoietic cell population was performed using CD34 UltraPure human MicroBeads Kit and a Mini (MS) & Midi (ML) MACS Starting Kit, as in the manufacturer's protocol.

Intracellular immunostaining

Adherent cells on 48-well plates were fixed and permeabilized by 15 min incubation at room temperature with 4% Paraformaldehyde and 0.5% Triton solution. Non-specific binding of proteins to the antibody was blocked with 10% FCS Perm/Wash buffer. HA-RUNX1-ETO was detected using an Anti-HA-tag primary conjugated antibody and nuclei were stained with DAPI. Cells were subsequently analyzed by epifluorescence imaging.

Imaging

Epifluorescence images of the *in vitro* hematopoietic cultures and immunostainings were taken using the 10x and 20x objectives of a Zeiss AxioObserver Z1 microscope and a Zeiss AxioCam monochrome camera and were processed with the Zen Blue software. Confocal images of the *in vitro* hematopoietic cultures were taken with a Zeiss LSM780 microscope using a 10x objective and processed with Zen Black software. All images were exported as separate layers in JPEG format and assembled in Adobe Photoshop when required. Brightness and contrast adjustments were applied equally to all images.

Colony-forming assays

Colony-forming-unit (CFU) assays were performed as reported (Ng et al., 2016) with some modifications. Briefly, 3–5 × 10^3 cells were cultured in 1% methylcellulose, supplemented with the '5-factor' cytokine mix (described in hematopoietic differentiation section) plus 10 μg/ml hLDL and 5 U/ml EPO. For the preparation of 1% methylcellulose, 40 mL serum-free 2.6% MethoCult^{TM} H4100 was mixed with an equal volume of 2x STAPEL-P medium (STAPEL medium made with IMDM containing 2x supplements and

without PFHMII) plus 20 mL of 1x STAPEL medium to give a final volume of 100 ml. Cells were cultured either with or without Dox and each condition was set up in triplicates in ultra-low attachment 24-well plates. Plates were scored for hematopoietic CFUs after 7 to 10 days.

Replating assays

Replating assays were conducted on non-adherent hematopoietic progenitors plated at a known concentration on Matrigel-coated 6-well plates. Cells were harvested, counted and replated weekly. Live and death cells were determined using a FL Countess-II Automated Cell Counter (Thermo Fisher Scientific) after Trypan-Blue staining.

Cell cycle analysis

Cells in culture were incubated for 3 h with 25 μM BrdU and non-adherent progenitor cells were fixed in 75% ethanol. Suspensions of fixed cells were pelleted and re-hydrated with PBS for 20 min and double stranded DNA was subsequently denatured by 20-minute incubation with 200 μL 2N HCl. Cells were washed twice with PBS and twice with blocking buffer (5% FBS, 0.1% NaN_3, 0.1% TritonC100 in PBS). Samples were subjected to RNaseA treatment (100 μg/ml) in PBS for 30 min at 37°C and both IgG control and BrdU stainings were performed using FITC-conjugated antibodies at room temperature for 50 min. Cells were washed with PBS and incubated for 30 min with 1 μM Vybrant DyeCycle Violet Stain in PBS prior to flow cytometric analysis.

Western Blotting

Nuclear protein extracts were prepared from progenitor cells floating in culture using the Universal Magnetic Co-IP Kit and quantified using the Pierce BCA Protein Assay Kit, following the manufacturer's protocol. Protein samples were diluted to the same amount, denatured at 95°C for 10 min and run on a 4%–20% gradient polyacrylamide gel. Proteins in the gel were then transferred to a nitrocellulose membrane using the mixed protocol in a Transblot Turbo (BioRad). The membrane was blocked with 5% milk in Tris Buffered Saline Tween (TBST) prior to hybridization with primary antibody (anti-AML1, 1:1,000 diluted in 5% milk TBST) overnight at 4°C. The membrane was then washed with TBST and incubated with an anti-rabbit horseradish peroxidase-conjugated secondary antibody (1:10,000 in 5% milk TBST) for 1h at room temperature. The membrane was then subjected to TBST washes and enhanced chemiluminescence was detected in a developer following incubation with SuperSignal PICO reagent mix. Nuclear loading control was performed using an anti-H3 antibody (1:10,000).

Gene expression analysis

Total RNA was extracted using the Bioline Isolate II RNA Mini Kit according to the manufacturer's instructions. cDNA was reverse-transcribed using random hexamer priming and Tetro cDNA synthesis kit or using Oligo $(dT)_{18}$ priming and SuperScript II Reverse Transcriptase, according to the manufacturer's guidelines. Gene expression was analyzed by quantitative real-time PCR analysis using Taqman reagents and probes or SYBR Green master mix and primers designed to amplify cDNA fragments. All probes and primers are listed in Key Resources Table. Analyses were performed in technical duplicates and GAPDH was used as the reference gene to normalize data.

RNA-Seq library preparation

RNA-sequencing (seq) libraries were prepared using a TruSeq® Stranded mRNA Library Prep following the Low Sample (LS) workflow according to manufacturer's instructions. Libraries were subjected to a quality control using a High Sensitivity DNA chip on an Agilent Technologies 2100 Bioanalyser instrument and were quantified using the RT-qPCR-based method KAPA Library Quantification Kit for Illumina Sequencing Platforms, following the manufacturer's protocol. Libraries were run in a pool of twelve indexed libraries in a NextSeq (Illumina) machine using sequencing by synthesis chemistry and a NextSeq® 500/550 High Output 150 cycle sequencing kit v2, obtaining 75 bp paired-end reads.

Assay for transposase-accessible chromatin with high-throughput sequencing (ATAC-seq)

Chromatin accessibility was evaluated by ATAC-seq using a modified protocol to as reported (Buenrostro et al., 2015; Corces et al., 2016). Briefly, 50,000 single-cell sorted hematopoietic progenitors were pelleted and snap-frozen upon resuspension in 5 μL sucrose freezing buffer, consisting of 60 mM KCl, 15 mM NaCl, 5 mM $MgCl_2$, 10 mM Tris pH 7.4 and 1.5 M sucrose. Transposition reaction was performed for 30 min at 37°C upon addition of 45 μL of ATAC reaction mix, consisting of 25 μL Tagmentation DNA Buffer and 2.5 μL Tn5 Transposase enzyme (both from the Nextera DNA Library Prep Kit), 1 μL of 0.5% Digitonin and 16.5 μL water. DNA was purified using a MinElute Reaction Cleanup Kit and DNA fragments were subsequently amplified using Customized Nextera PCR Primer Adaptors and NEBNext High-Fidelity 2x PCR Master Mix. Optimal number of cycles, prior reaching saturation of the PCR in order to reduce GC and size bias, was determined by monitoring the reaction as previously described (Buenrostro et al., 2015). Adaptor dimers were cleaned up from the libraries using AMPure magnetic beads prior to validation. Libraries were evaluated using a High Sensitivity DNA chip on an Agilent Technologies 2100 Bioanalyser instrument and concentration was measured using a KAPA Library Quantification Kit. Libraries were also validated by RT-qPCR evaluation of the ratio of open (TBP promoter) to closed regions of DNA (chromosome 18) and active gene body (β-actin). Libraries were sequenced in a pool of twelve indexed libraries in a NextSeq (Illumina) machine and a NextSeq® 500/550 High Output 75 cycle sequencing kit v2, obtaining 75 bp single-end reads, at the Genomics Birmingham sequencing facility.

Chromatin Immunoprecipitation with high-throughput sequencing (ChIP-Seq)

All ChIP experiments were performed after single crosslink with 1% methanol-free formaldehyde for 10 min, as reported (Obier et al., 2016). RUNX1, RUNX1-ETO (HA tag), LMO2, and LDB1 ChIP experiments (0 and 5 ng/ml Dox samples) were performed on CD34+ magnetic sorted progenitors. H3K4me3, H3K27ac (0 and 5 ng/ml Dox samples) and RUNX1 and RUNX1-ETO (HA tag) (0, 5 and 10 ng/ml Dox samples) were performed on non-adherent mixed progenitors (> 30% CD34+). Antibodies used are listed in Key Resources Table. ChIP libraries for Illumina sequencing were prepared using the KAPA Hyper Prep Kit, as detailed by the manufacturer. Quality control of the libraries was performed using a High Sensitivity DNA chip on an Agilent Technologies 2100 Bioanalyser instrument and libraries were quantified using a KAPA Library Quantification Kit. Libraries were sequenced in a pool of twelve indexed libraries in a NextSeq (Illumina) machine and a NextSeq® 500/550 High Output 75 cycle sequencing kit v2.

Single Cell RNA-Seq (scRNA-Seq)

Non-adherent progenitors at day 22 of differentiation (untreated and upon 24-hour Dox treatment) were sorted for CD45+ CD34+ and RUNX1C+. Cells were re-suspended in 80 μL at a concentration of 100-1200 cells/μl for evaluation of cell viability prior to loading of 4000 single cells on a Chromium Single Cell Instrument (10X Genomics). Library generation for scRNA-seq was performed by the Genomics Birmingham Sequencing Facility using the Chromium Single Cell 3′ Library and Gel Bead Kit v2. Libraries were paired-end sequenced on an Illumina NextSeq machine using the cycle parameters recommended by 10X Genomics.

QUANTIFICATION AND STATISTICAL ANALYSIS

Statistical analysis

Experiments were analyzed using GraphPad Prism versions 5–7 (GraphPad Software Inc.) and Microsoft Excel (Microsoft corporation).

Bulk RNA-Seq data analysis

Sequencing adaptors and low quality bases were trimmed from the raw RNA-Seq reads using Trimmomatic v0.32 (Bolger et al., 2014). The processed reads were then aligned to the human genome (version hg38) using Hisat2 v2.1.0 (Kim et al., 2015) with default settings. Gene expression was measured as fragments per kilobase of transcript per million mapped reads (FPKM) values using with Stringtie v.1.3.3 (Pertea et al., 2015) with default settings. Gene models from the RefSeq database (O'Leary et al., 2016) were used as the reference transcriptome. Only genes that were expressed with an FPKM > 1 in at least one of the samples were retained for further analysis. The raw FPKM values were quantile normalized using the Limma package v3.26.9 (Ritchie et al., 2015) in R v3.5.1. The normalized data were then log2-transformed, with a pseudocount of 1 being added to each of the FPKM values prior to transformation. Differential gene expression analysis was carried out using Limma. A gene was considered to be differentially expressed if it had a greater than 2-fold change between experimental conditions, and a Benjamini-Hochberg adjusted p value < 0.05. Kyoto encyclopedia for genes and genomes (KEGG) pathway enrichment analysis was done using the ClueGO plugin v2.5.0 (Bindea et al., 2009) for Cytoscape v.3.61 (Shannon et al., 2003). This was done using a right-sided hypergeometric test, with Benjamini-Hochberg p value correction for multiple testing. A pathway was deemed to be significantly enriched if the adjusted p value was < 0.05. Hierarchical clustering of RNA-Seq samples and replicates was done by first calculating the Pearson correlation value for each pair of samples. The resulting correlation matrix was then hierarchically clustered using complete linkage clustering of the Euclidean distances, and finally plotted as a heatmap in R. To carry out gene expression co-variance analysis, gene expression values were first transformed to Z-scores using the scale function in R. These were then hierarchically clustered using complete linkage of the Euclidean distances. Clusters corresponding to sets of genes with similar patterns of expression were then extracted from the dendrogram using the dynamicTreeCut package v1.63 (Langfelder et al., 2008) in R using the hybrid method with a minimum cluster size of 25 genes. To compare the gene expression profile of the RUNX1-ETO induced cells to that of AML patients with the t(8;21) translocation, RNA-Seq data from t(8;21) patients and from healthy peripheral blood stem cells (PBSCs) from Assi et al. (2019) was downloaded from GEO using the accession GEO: GSE108316. These data were aligned and processed as described above. The sets of genes that were up and downregulated in the RUNX1-ETO induced cells was then compared to the gene expression profiles of the t(8;21) AML cells and PBSCs using gene set enrichment analysis (GSEA) using the GSEA software (Subramanian et al., 2005).

ATAC-Seq data analysis

Single-end reads from ATAC-Seq experiments were processed to remove low-quality bases and sequencing adaptors using Trimmomatic. Reads were then aligned to the human genome (version hg38) using Bowtie2 v2.2.6 (Langmead and Salzberg, 2012) with the parameter–very-sensitive-local. Reads that aligned to the mitochondrial genome were removed from further analysis. Potential PCR duplicated reads were identified and removed from the alignments using Picard v2.10.5 (http://broadinstitute.github.io/picard). Open chromatin regions (peaks) were identified using MACS2 v2.1.1 (Zhang et al., 2008) using the settings–nomodel–nolambda -B–trackline. The resulting peaks were then filtered against the hg38 blacklist and simple repeat tracks from the UCSC table browser (Karolchik et al., 2004) to remove any potential artifacts. Peaks were annotated to the nearest gene, and then further annotated as either a promoter of distal element using the annoatePeaks.pl function in the Homer software package v4.9.1

(Heinz et al., 2010). A peak was annotated as being within a gene promoter if it was within 1.5kb of a transcription start site (TSS) and as a distal element otherwise. ATAC peak unions were constructed by merging peaks that had summit positions within 400 bp of each other. In these cases, peaks were combined to a single peak with a new summit position defined as the mid-point between the summit positions of the original peaks. These average peak positions were used in all further downstream analysis. To identify regions of differential chromatin accessibility, a peak union was first created for each pair of samples being considered. The read density for these peaks was then retrieved directly from the bedGraph files produced by MACS2 using the annotatePeaks.pl function in Homer with the parameter -size 200. These tag counts were normalized as counts per million (CPM) in R, and further log2-transformed with a pseudocount of 1 added to each value prior to transformation. A peak was considered to be differentially accessible if the fold-difference of the normalized tag count was greater than 2 between experiments. Motif enrichment analysis was then carried out in these sets of peaks using the findMotifsGenome.pl function in Homer. To create read density plots, peaks were first ordered according to fold-difference. The read density in a 2kb window centered on the peak summits was then calculated using from the bedGraph files produced by Homer using the annotatePeaks.pl file in Homer, using the options -size 2000 -hist 10 -ghist. These were then plotted as heatmaps using java TreeView v1.1 (Saldanha, 2004). ATAC-Seq data from hematopoietic cell type in various stages of differentiation were obtained from Corces et al. (2016) via GEO using the accession GEO: GSE74912. These data were aligned and processed as described above.

ChIP-Seq data analysis

Reads from ChIP-Seq experiments were processed, aligned to the human genome and de-duplicated in the same way described above for the ATAC-Seq data. Peaks from ChIP-Seq experiments targeting the transcription factors RUNX1, RUNX1-ETO, LDB1 and LMO2 were identified using MACS2 with default settings. These peaks were then compared to the ATAC-Seq data, with only peaks that occurred within open chromatin regions being retained for further analysis. To identify differential binding of RUNX1 between the 0 and 5 Dox datasets, a union of RUNX1 peaks was first constructed by merging peaks that had summits within 100 bp of each other. The read density in these peaks was then retrieved using the annoatePeaks.pl function in Homer and normalized as counts per million in R. Peaks that had a fold-difference of at least 2 were considered to be differentially bound between experiments. RUNX1 and RUNX1-ETO target genes were identified by annotating each peak to its closest TSS using the annotatePeaks.pl function in Homer. Peaks corresponding to the histone modifications H3K27ac and H3K4me3 were called using MACS2 with default settings. These peaks were then filtered against the hg38 blacklist and simple repeat tracks from the UCSC table browser to remove any potential artifacts.

Construction of average profiles

Average profiles for ATAC and ChIP-Seq data were constructed by first normalizing each of the alignment tracks as counts per million (CPM) using the bamCoverage function in deepTools v3.2.0 (Ramírez et al., 2016). These were then plotted using the plotProfile function in deepTools.

Single cell RNA-Seq data analysis

Illumina base call (BCL) files that were generated using the Chromium platform from 10x genomics were de-multiplexed and converted to the fastq format using the mkfastq function in CellRanger v2.1.1. These were then aligned to the human genome (version hg38) using the count function in CellRanger. Gene models from the RefSeq database were used as the reference transcriptome. Unique molecular identifier (UMI) counts were processed and normalized using the Seurat v2.3.4 package (Butler et al., 2018) in R. Cells with less than 1500 detectable genes, or that had more than 10% of UMIs aligned to mitochondrial genes were removed from further analysis. Additionally, genes that were detected in less than 20 cells were also excluded from analysis. The cell cycle stage for each cell was inferred using the CellCycleScoring function in Seurat. The possible effects of cell cycle stage, as well as sequencing depth (as measured by the total number of UMIs) per cell were removed from the analysis by linear regression using the ScaleData function in Seurat. Clustering of cells was performed by first combining the datasets from the 0 and 5 dox treated cells into a single dataset using canonical correlation analysis (CCA). This combined dataset was then clustered using the t-distributed stochastic neighbor embedding (t-SNE) method. Cell clusters were identified using the FindClusters function in Seurat, using a resolution value of 0.4. Cell marker genes, corresponding to genes that are enriched on one cluster relative to others, were identified using the FindMarkers function. A gene was considered as a marker gene if it had a log fold-change value greater than 0.5 and could be detected in at least 50% of cells in that cluster. Differential gene expression analysis was also carried out for each cluster using the FindMarkers function, with genes with a log-fold-change greater than 0.25 and an FDR < 0.05 being considered to be differentially expressed. Cell trajectory (pseudotime) analysis was carried out using Monocle v2.10.1 (Qiu et al., 2017; Trapnell et al., 2014). Normalized UMI counts from Seurat were first imported into Monocle using the importCDS function. Cells were then ordered along a pseudotime trajectory using the discriminative dimensionality reduction with trees (DDRTree) method using the complete set of cell marker genes identified by Seurat to order the cells.

Cell Reports, Volume *31*

Supplemental Information

Expression of RUNX1-ETO Rapidly Alters the Chromatin Landscape and Growth of Early Human Myeloid Precursor Cells

Monica Nafria, Peter Keane, Elizabeth S. Ng, Edouard G. Stanley, Andrew G. Elefanty, and Constanze Bonifer

Figure S1

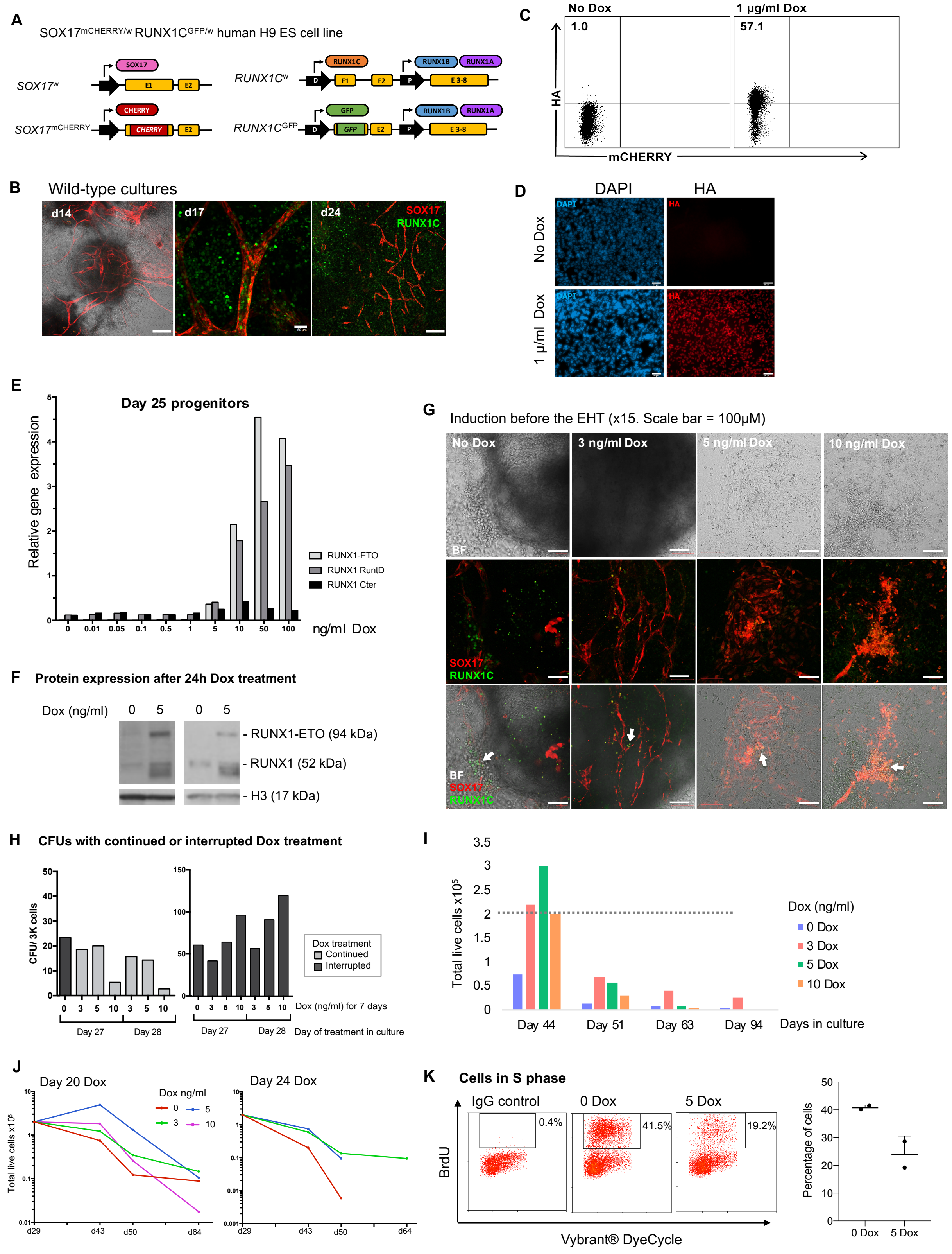

FIGURE S1 (Related to Figures 1 and 2). Expression of *RUNX1-ETO* leads to a reversible differentiation and growth arrest of human early hematopoietic progenitor cells

A) Schematic representation of the targeted alleles in the SOX17$^{mCHERRY/w}$ RUNX1C$^{GFP/w}$ human H9 ES dual reporter cell line. Left: Wild-type and targeted alleles in the *SOX17* locus, with mCHERRY sequence inserted into exon 1. Right: Wild-type and targeted alleles in the *RUNX1* locus. GFP sequence was inserted into exon 1, resulting in expression of GFP from the distal (D) promoter and RUNX1B and RUNX1A isoforms from the proximal (P) promoter within the same allele. Promoters and exons are shown with black arrows and yellow boxes, respectively. Protein products generated from each allele are represented with ovals.

B) RUNX1C+ hematopoietic progenitors emerge from cell clusters located within vascular structures of SOX17+ hemogenic endothelium after the EHT in wild-type EB cultures. Confocal images of EB cultures from the SOX17$^{mCHERRY/w}$ RUNX1C$^{GFP/w}$ human H9 ES double reporter cell line at several days during differentiation, as indicated. *SOX17* (mCHERRY) expression marks arterial structures and *RUNX1C* (GFP) marks hematopoietic progenitors. Scale bar: 200 µm (d14, d24), 50 µm (d17). Fluorescence and brightfield channels are merged at d14 image. At d14: EBs appear as opaque round structures surrounded by a stromal layer containing SOX17+ hemogenic endothelium and arterial structures. At d17: RUNX1C+ blood progenitors are generated from SOX17+ hemogenic endothelium, mimicking structures observed during embryonic AGM hematopoiesis. At d24: progenitors have detached from the endothelium and gone in suspension to further grow and differentiate.

C) Intracellular flow cytometry analysis showing RUNX1-ETO induction upon addition of 1 µg/ml Dox for two days in a pooled population of puromycin-resistant cells after transfection. Detection using an anti-HA tag DyLight® 650-conjugated antibody.

D) Immunofluorescence assay showing RUNX1-ETO induction upon addition of 1 µg/ml Dox for two days in a single-sorted clone. Images are of clone #18 and representative of six single-sorted clones. Cell nuclei are stained with DAPI (blue) and cells expressing HA-*RUNX1-ETO* are detected with an anti-HA antibody (red). Fluorescence channels are merged on right panels. Scale bar: 50 µm.

E) *RUNX1* and *RUNX1-ETO* gene expression in response to Dox treatment for 24h on d25 hematopoietic progenitors. Primers were designed to amplify: the translocation breakpoint (RUNX1-ETO), the DNA-binding domain present in both gene products (RUNX1 RuntD) and the carboxy-terminal domain present only in endogenous RUNX1 (RUNX1 C-ter). Gene expression was normalized to that of GAPDH.

F) RUNX1 and RUNX1-ETO protein expression measured by Western Blotting in nuclear extracts from floating progenitors uninduced (0 Dox) and induced with Dox (5 ng/ml) at day 21 for 24h. The figure shows two biological replicates. RUNX1 and RUNX1-ETO were detected using the same antibody, which binds to the RUNX1 homology domain. H3 protein expression was measured as a loading control.

G) RUNX1-ETO induction before the EHT disrupts the vascular organization and disrupts blood formation. Confocal images of combined Z-stack layers from d16 hematopoietic differentiation cultures with RUNX1-ETO induced from d10 (before the EHT) using 3, 5 or 10 ng/ml Dox. White arrows in the 0 Dox condition indicate emerging RUNX1C-/+ blood progenitors. Arrows in the Dox-treated samples indicate aberrant phenotypes including reduced numbers of emerging progenitors (3 ng/ml Dox), disorganized vasculature (5 ng/ml Dox) and progenitors co-expressing *RUNX1C* and *SOX17* (10 ng/ml Dox). Brightfield (top), fluorescence (middle) and merged field channels (bottom) are shown. Scale bars: 100 µm. SOX17 (mCHERRY, red) and RUNX1C (GFP, green).

H) RUNX1-ETO causes a reversible proliferation block (Related to Figure 2A). Colony-forming unit (CFU) assays of definitive progenitors from EB cultures treated with Dox during 7 days at day 27 and day 28. For the CFU assays, progenitors were plated in methylcellulose in triplicate at a concentration of 3,000 live cells/well in either presence (continued treatment, light grey) or absence (interrupted treatment, dark grey) of Dox. Individual graphs correspond to different biological replicates.

I) Previously induced progenitor cells show an initial growth response and increased survival compared to uninduced cells (Related to Figure 2B). EB cultures were treated at d22 with 0, 3, 5 or 10 ng/ml Dox for 7 days and floating progenitors were harvested and subjected to serial replating assays with continued Dox treatment. The graph displays the cell growth data represented in Figure 2B in absolute values ($x10^5$) of the total live cells. The dotted line represents the number of progenitors plated at the start of the replating assay.

J) Low levels of RUNX1-ETO induction increases the survival of a subset of progenitor cells. Additional examples to data shown in Figure 2B. Replating assays of hematopoietic progenitors from cultures treated at d20 or d24 with different Dox concentrations, showing two representatives each of three independent experiments. Floating hematopoietic cells were plated at 2 $x10^5$ cells/well in the correspondent Dox concentration and cell numbers were measured weekly at three time points, as indicated. On d24 graph, only 3-dox treated cells were able to survive over 28 days in the replating assays.

K) RUNX1-ETO produces a cell cycle arrest in the G1 phase. Flow cytometry plots showing cell cycle kinetics of wild-type and RUNX1-ETO-induced progenitor cells. EB cultures were induced at d21 with 5 ng/ml Dox for 4 days and then were pulse-labelled with 25 µM BrdU for 3.5h. Non-adherent cell progenitors were fixed and stained with FITC-conjugated anti-BrdU antibody and Vybrant-DyeCycle. DNA content and cell cycle distribution were analyzed by flow cytometry. Boxed cells represent cells that have entered the S-phase of the cell cycle during the BrdU incubation. FITC IgG control is shown. Graph on the right shows the percentage of cells in S-phase corresponding to two biological replicates (represented as dots) and the median values (represented with a line).

Figure S2

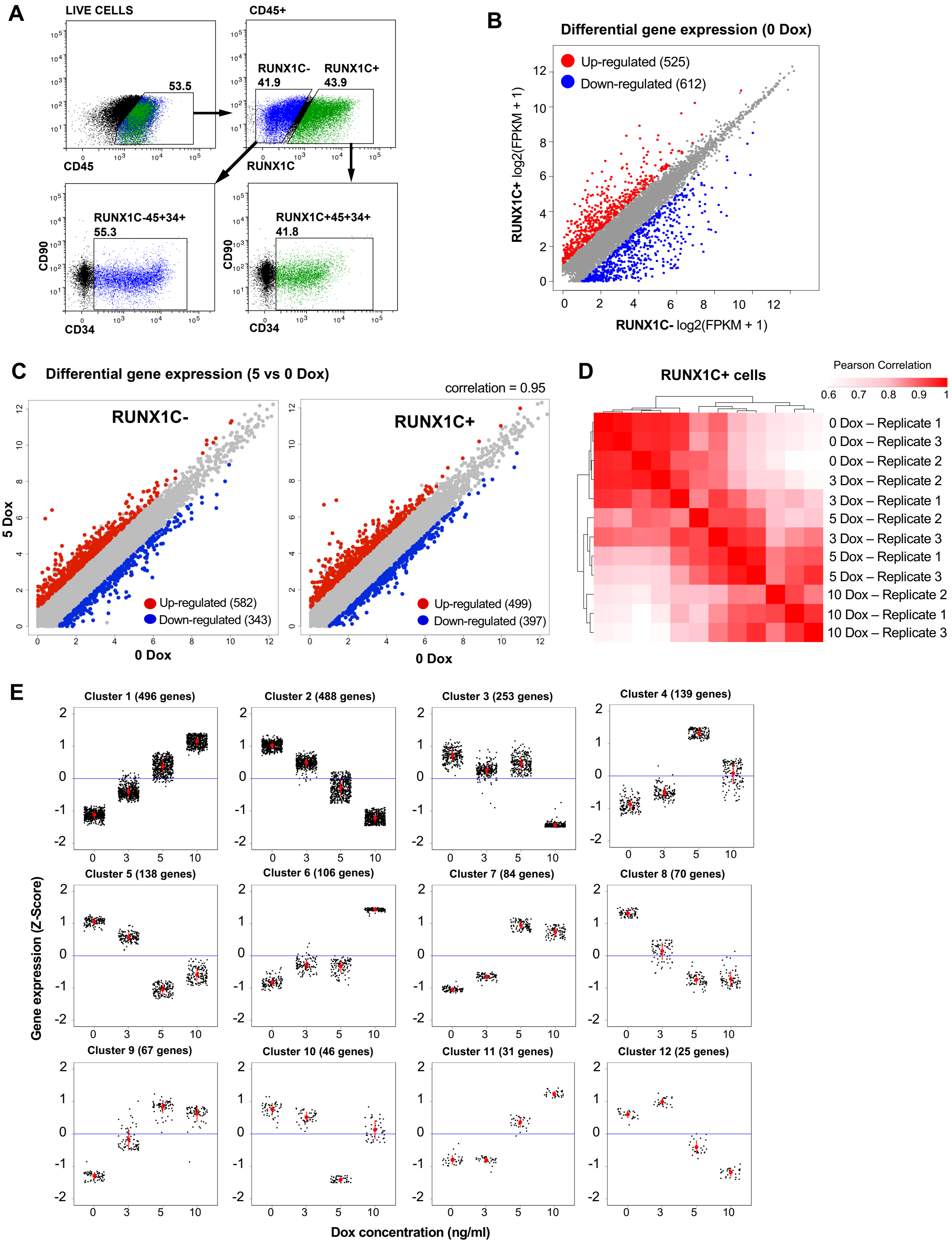

FIGURE S2 (Related to Figure 3). RUNX1-ETO induction leads to cell-type and dose-dependent changes in gene expression

A) Flow cytometry strategy for sorting of d21 cultures based on CD45 (BV), RUNX1C (GFP/FITC) and CD34 (Pe-Cy7) expression.

B) Clustering of gene expression RNA-Seq data by log2 fold FPKM +1 (fragments per kilobase of transcripts per million mapped reads) values of genes differentially expressed (two-fold change) after RUNX1-ETO induction using 5 ng/ml Dox in both RUNX1C- and RUNX1C+ (CD45+ CD34+) populations. Adjusted P value <0.05.

C) Clustering of gene expression RNA-Seq data by log2 fold FPKM +1 (fragments per kilobase of transcripts per million mapped reads) values of genes differentially expressed (two-fold change) after RUNX1-ETO induction using 5 ng/ml Dox in both RUNX1C- and RUNX1C+ (CD45+ CD34+) populations. Adjusted P value <0.05.

D) Hierarchical clustering using Pearson correlation coefficients of gene expression in CD45+ CD34+ RUNX1C+ progenitors upon 24-hour Dox exposure (0, 3, 5 or 10 ng/ml) from three biological replicates.

E) Co-variance analysis of gene expression RNA-Seq data by Z-score from CD45+ CD34+ RUNX1C+ sorted progenitor cells upon RUNX1-ETO induction with 3, 5 or ng/ml Dox for 24h, showing 12 clusters/groups of genes with differential expression response to the level of RUNX1-ETO induction. Number of genes comprised on each cluster are indicated. Black dots represent transcript levels for each individual gene. Red dots and bars represent the mean and standard deviation.

Figure S3

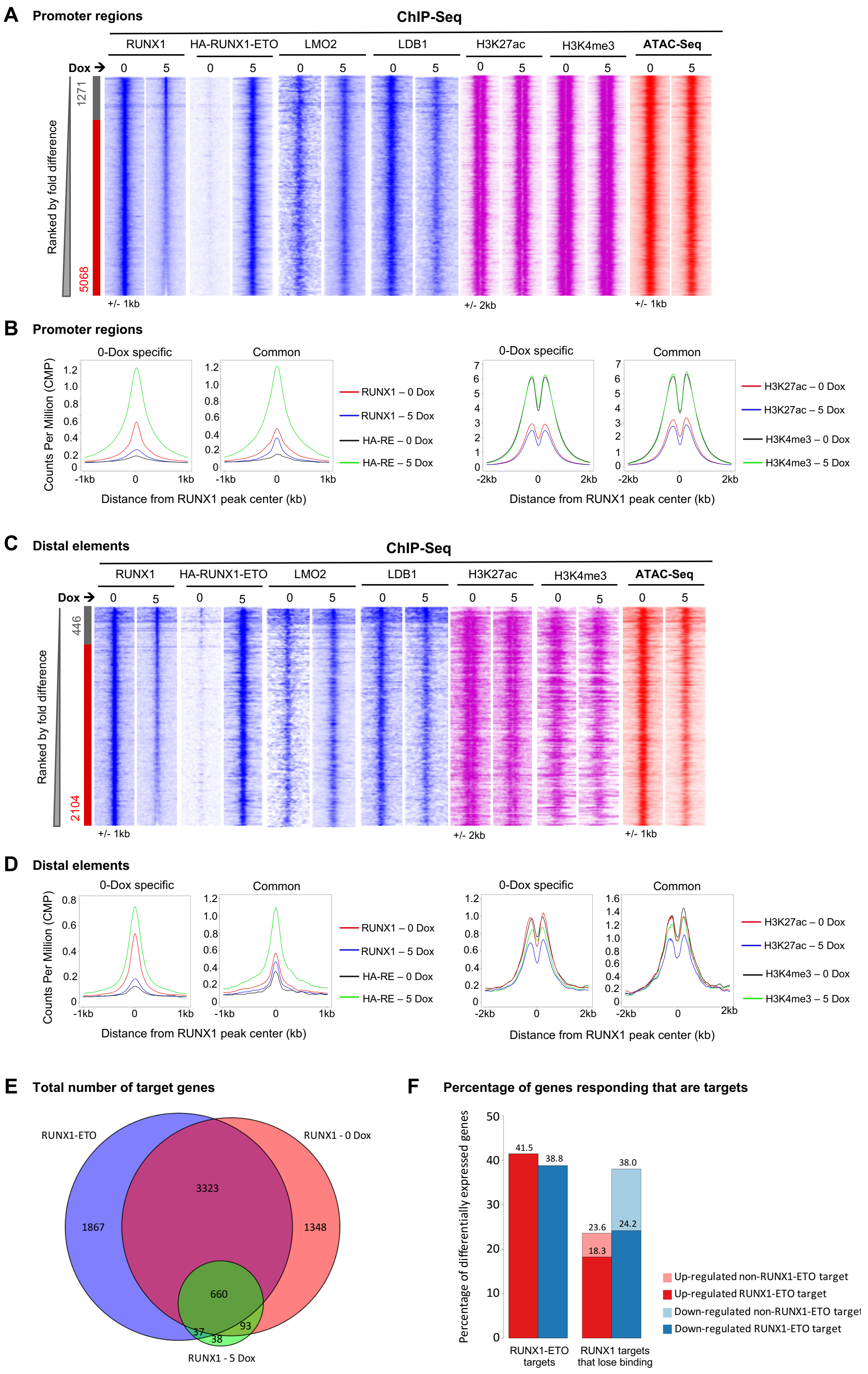

FIGURE S3 (Related to Figures 4 and 5). RUNX1-ETO blocks the binding of RUNX1 at distal elements and leads to down-regulation of RUNX1 (but not RUNX1-ETO) target genes

A and C) Comparison of RUNX1 binding at promoter (A) and distal (C) regions from ChIP-Seq in 0 and 5 Dox-treated CD34+ populations ranked by fold difference, considering peaks with enrichments greater than 2-fold between samples to be specific. Sample-specific sites and number of peaks are indicated alongside, being: red the 0-Dox specific and grey the shared peaks. ChIP-Seq enrichment for (HA)-RUNX1-ETO, LMO2, LDB1, H3K27ac and H3K4me3 in each sample and chromatin accessibility (ATAC-Seq) peaks are plotted along the same coordinates as the RUNX1 ChIP-Seq promoter (A) and distal element (C) peaks.

B and D) Average profiles for transcription factor (Top panels) and histone modification (Bottom panels) ChIP-Seq data centered on RUNX1 promoter (B) or RUNX1 distal element (D) -binding peaks in the 0 Dox-specific and common peaks.

E) Venn diagram of RUNX1-ETO and RUNX1 ChIP data showing overlap of binding events between the total number of genes targeted by (i) RUNX1-ETO, (ii) RUNX1 0 Dox (in uninduced cells) and (iii) RUNX1 after 5 Dox induction.

F) Graph depicting the percentage of differentially expressed (up- or down-regulated) genes that respond to RUNX1-ETO induction and are either RUNX1-ETO or RUNX1 targets.

Figure S4

A

Sequencing statistics - 0 Dox	
No. of cells sequenced	3,327
No. of cells passed QC	3,141
Median no. of reads per cell	75,452
Median no. of genes per cell	3,149

Sequencing statistics - 5 Dox	
No. of cells sequenced	3,814
No. of cells passed QC	3,669
Median no. of reads per cell	67,098
Median no. of genes per cell	3,166

B

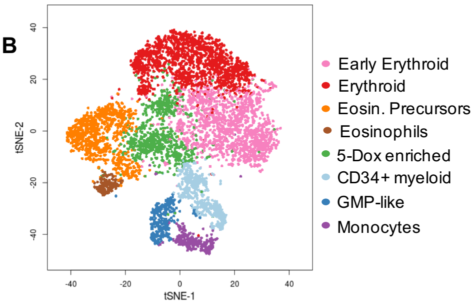

C

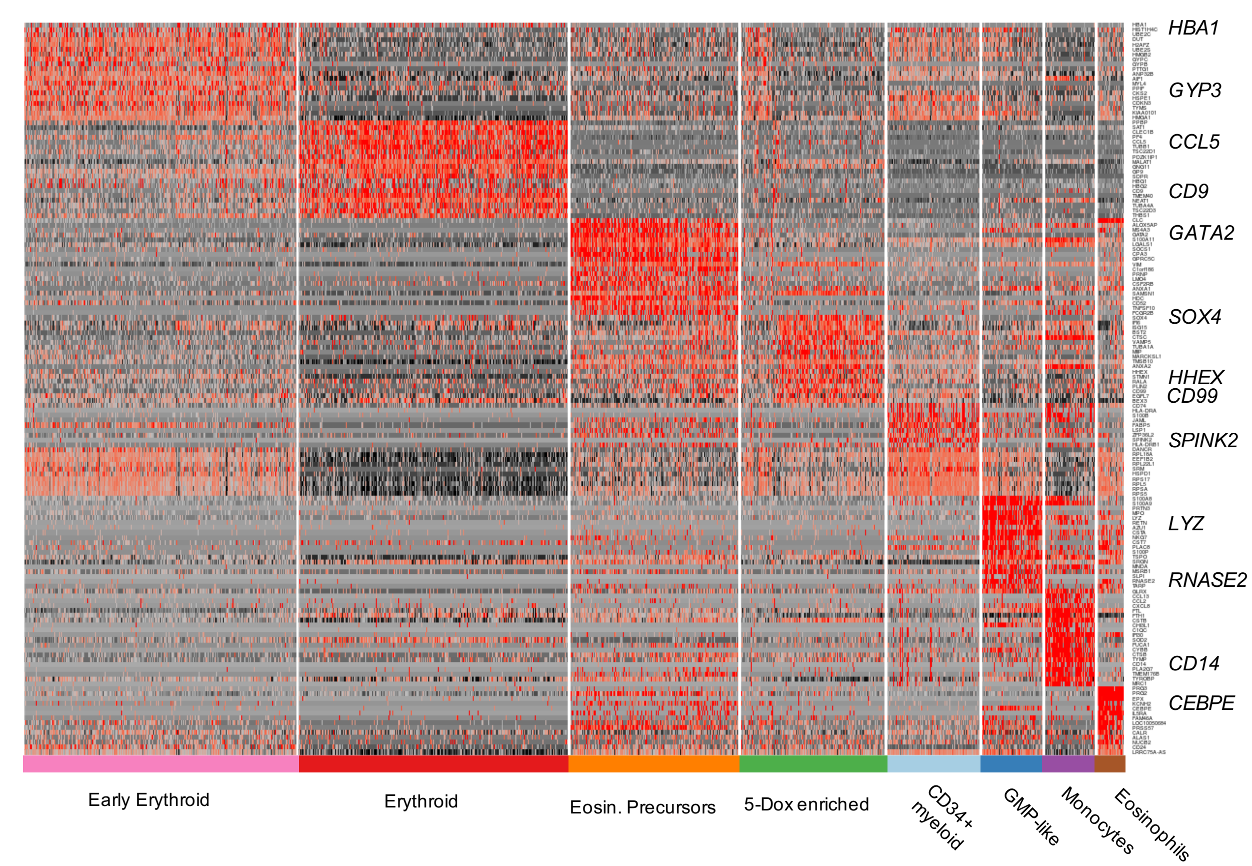

D

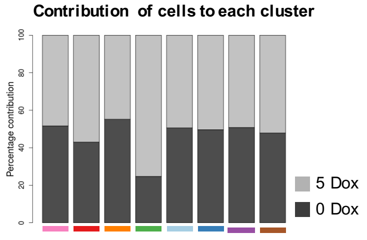

E

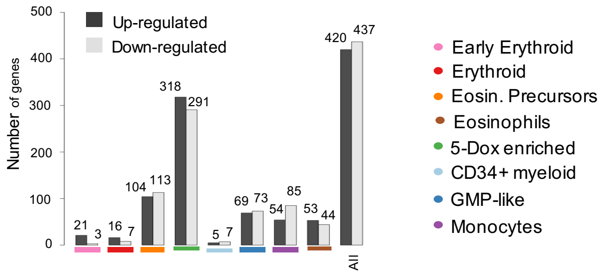

FIGURE S4 (Related to Figure 6). The CD45+CD34+RUNX1C+ population contains precursors from distinct blood lineages as well as multipotent cell progenitors

A) Table of the scRNA-Seq sequencing statistics showing the total number of sequenced cells, number of cells that passed the quality control (QC), and the median of reads and sequenced genes per cell for each 0 and 5 ng/ml Dox conditions.

B) Two-dimensional t-SNE maps displaying a total number of 7,135 CD45+ CD34+ RUNX1C+ sorted single cells from the combined data of 0 and 5 Dox treated cells, including identified cell populations based on expression of known marker genes.

C) Heatmap showing the expression of the top 20 marker genes specific to each cluster (same color coding as in (B)). Representative genes from each cluster are indicated.

D) Proportion bars showing the percentage of contribution of 0 and 5 ng/ml Dox dataset to each individual cell cluster.

E) Number of up- and down- regulated genes in each cell cluster upon treatment with 5 ng/ml Dox.

Figure S5

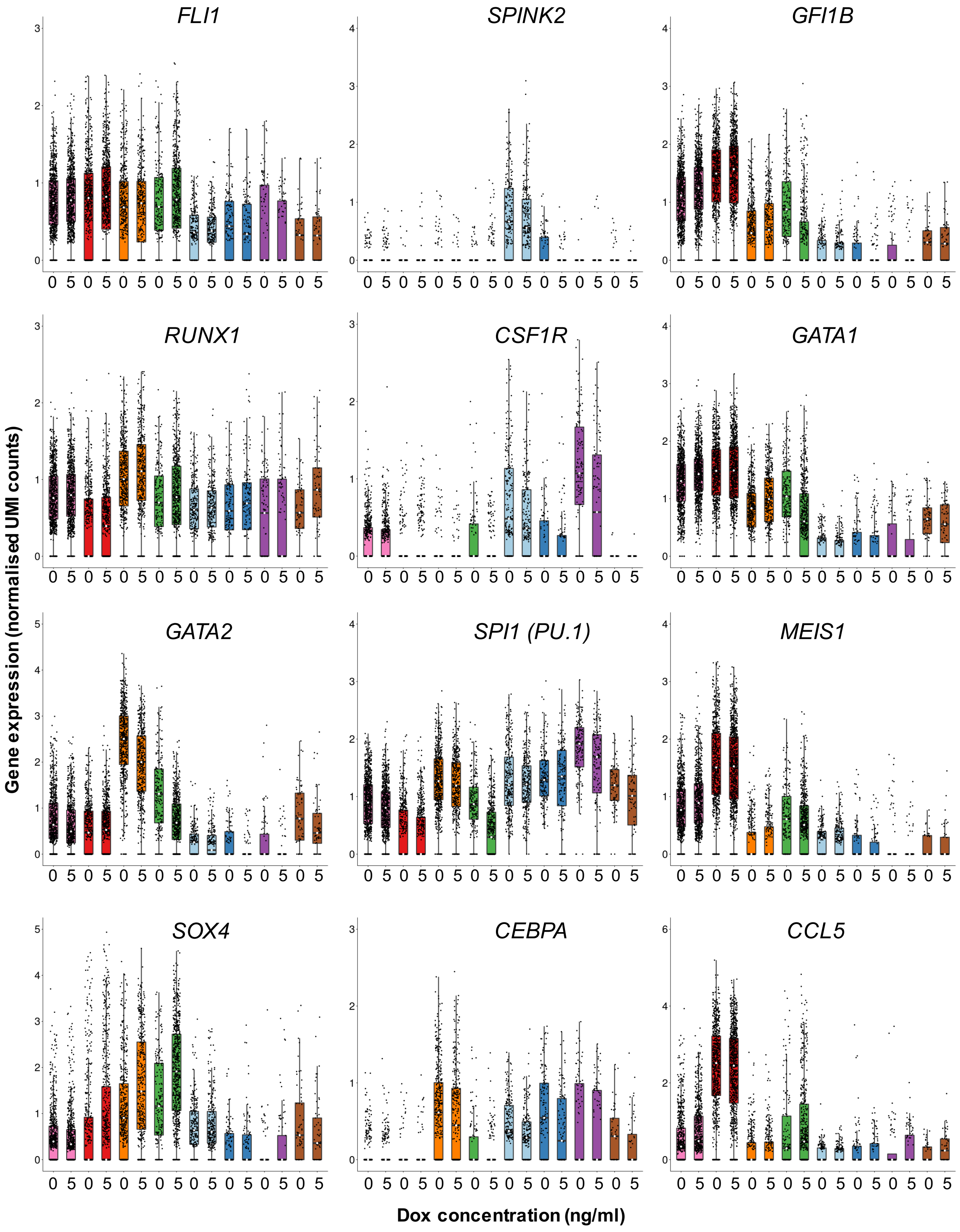

FIGURE S5 (Related to Figure 6). Induction of RUNX1-ETO results in the up-regulation of *SOX4* as well as down-regulation of important regulators of myelopoiesis in the 5-Dox enriched population

Box plots indicating expression levels of the individual marker genes represented in Fig 6D in the different ESC derived cell populations (color coded) and both datasets (0 and 5 Dox). Black dots represent transcript levels in an individual single cell. Boxes and bars represent the mean and standard deviation, respectively.

Figure S6

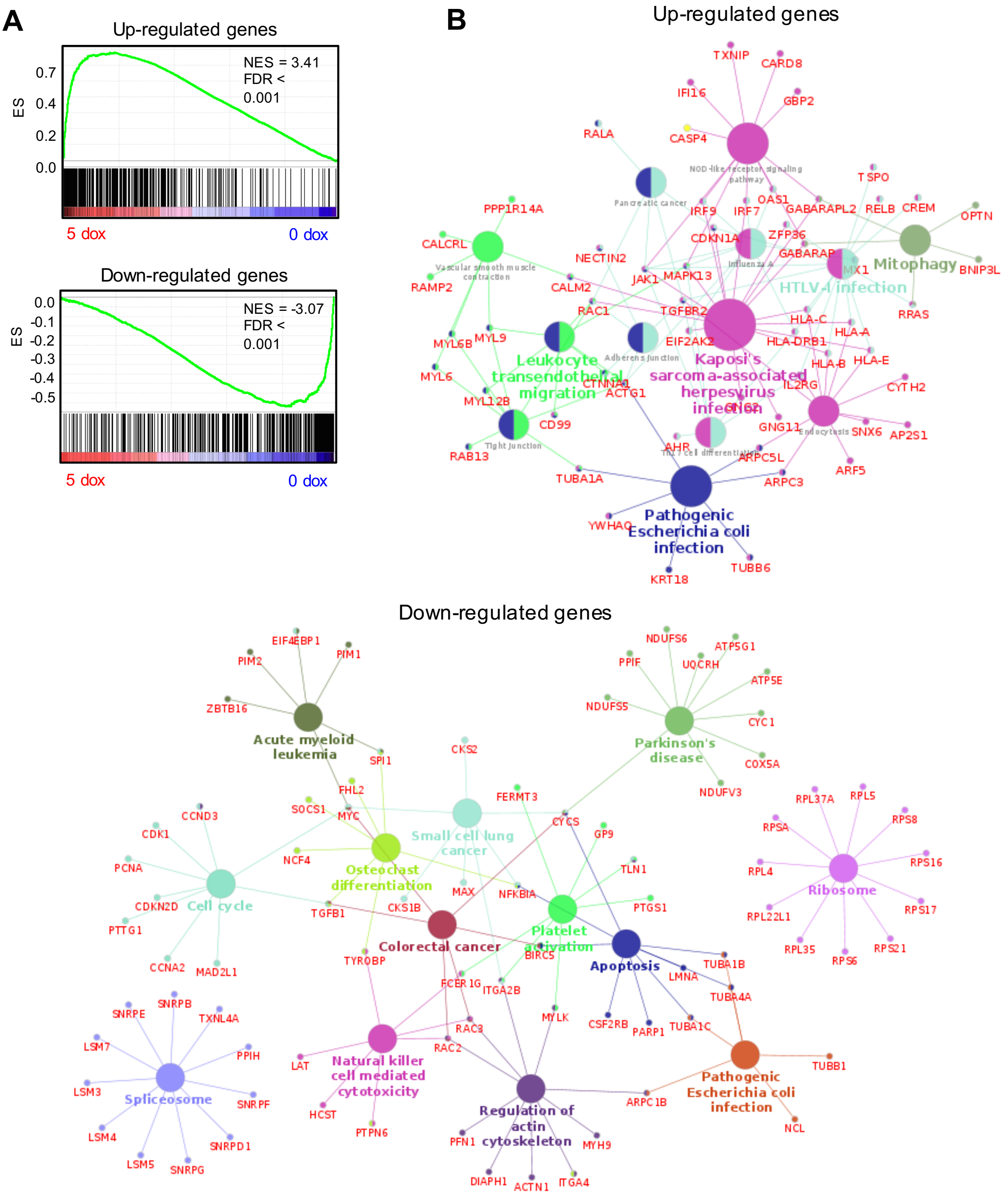

FIGURE S6 (Related to Figure 6). RUNX1-ETO induction yields a similar pattern of transcriptional dysregulation in the 5-Dox enriched and the 5 Dox-treated bulk progenitor populations

A) Gene Set Enrichment Analysis for correlation of up-regulated (top panel) and down-regulated (bottom panel) gene signatures between single-cell sorted cells and the CD45+ CD34+ RUNX1C+ bulk populations upon 5-Dox induction for 24 hours. ES, Enrichment Score; NES, Normalized Enrichment Score. FDR, False discovery rate.

B) Network diagram of KEGG pathways for up-regulated (above) and down-regulated (below) genes in the 5-Dox enriched cell cluster of CD45+ CD34+ RUNX1C+ sorted single cells upon 24-hours 5 ng/ml Dox treatment.

Oligonucleotides	Forward	Reverse
RUNX1-ETO cloning	TACCGTCGACCCGCCATGTACCCA TACGACGTCCCAGACTACGCTCGT ATCCCCGTAGATGCCAGCACGA	CGCAACGCGTCTACTAGCGAGGGGTTG TCTCTA
AAVS1 5' screen	GGACCACTTTGAGCTCTACT	TCCACGTCACCGCATGTTAG
AAVS1 3' screen	TGCCTGCTGACGCTCTTGACGATT	GAAGGATGCAGGACGAGAAA
Wild-Type screen	CCCCTATGTCCACTTCAGGA	CAGCTCAGGTTCTGGGAGAG
GAPDH	CCTGGCCAAGGTCATCCAT	AGGGGCCATCCACAGTCTT
RUNX1 C-ter	CCCTCAGCCTCAGAGTCAGAT	GGCAATGGATCCCAGGTAT
RUNX1 Runt Domain	AACAAGACCCTGCCCATCGCTTTC	CATCACAGTGACCAGAGTGCCAT
RUNX1-ETO junction	TCAAAATCACAGTGGATGGGC	CAGCCTAGATTGCGTCTTCACA

TABLE S1 (Related to Key Resources Table in STAR METHODS). List of primers generated in this study used for cloning, insertion of the transgene during cell line generation and gene expression analyses.
